# Structure and Properties of Poly(Ethylene-co-vinyl Acetate) Nanocomposites with Dual-Functionalized Dolomite Nanoparticles

**DOI:** 10.3390/ijms252312519

**Published:** 2024-11-21

**Authors:** Asfa Amalia Ahmad Fauzi, Azlin Fazlina Osman, Eid M. Alosime, Khairul Anwar Abdul Halim, Mohd Aidil Adhha Abdullah

**Affiliations:** 1 Faculty of Chemical Engineering & Technology, Universiti Malaysia Perlis (UniMAP), Arau 02600, Malaysia; asfauzi18@gmail.com (A.A.A.F.); kanwar@unimap.edu.my (K.A.A.H.); 2 Biomedical and Nanotechnology Research Group, Center of Excellent Geopolymer and Green Technology (CEGeoTech), Universiti Malaysia Perlis (UniMAP), Arau 02600, Malaysia; 3 King Abdulaziz City for Science and Technology (KACST), P.O. Box 6086, Riyadh 11442, Saudi Arabia; 4 Faculty of Science and Marine Environment, Universiti Malaysia Terengganu (UMT), Kuala Nerus 21030, Malaysia; aidil@umt.edu.my

**Keywords:** poly(ethylene-co-vinyl acetate), dual-functionalized dolomite nanoparticle, mechanical properties, thermal properties, biostability

## Abstract

Poly(ethylene-co-vinyl acetate) (PEVAc) is a copolymer that consists of non-polar polyethylene (PE) and a polar polyvinyl acetate (PVAc) monomer. PEVAc has high elasticity and is resilient, making it suitable for a variety of applications. However, the tensile strength of this copolymer needs to be improved for specific applications that require enough strength to tolerate high external tension or stress. This study proposed the use of dual-functionalized dolomite nanoparticles (DF-DNPs) composed of polar and non-polar nano-dolomite (P-DNPs and NP-DNPs) as nanofillers to reinforce the PEVAc. PEVAc/DF-DNP film appears to have a more homogeneous mixture, which is better for forming an optimal nanocomposite material. It also exhibits the highest tensile strength (10.48 MPa), elongation at break (1175.73%), and tensile toughness (62.12 MPa), which are higher by increments of 46.8%, 9.4%, and 20.3%, respectively, as compared to the neat PEVAc. The result proved that using DF-DNPs as a nanofiller can improve the strength of PEVAc while maintaining its flexibility to avoid brittleness of the nanocomposite film. Furthermore, its thermal characteristics were also successfully enhanced. A biostability assessment showed that the use of DF-DNPs as nanofiller caused the PEVAc copolymer to achieve the best water resistance, as it only exhibited a 2.63% weight increase, the lowest reduction in tensile properties among the studied fillers, and the best retention in surface degradation upon 3-month exposure to the in vitro environment. These findings indicate that the DF-DNPs help in developing a homogeneous nanocomposite by interacting with PE and PVAc.

## 1. Introduction

Polymers have been utilized worldwide since the introduction of this new area of study, causing the polymer industry to expand significantly. One of them is poly(ethylene-co-vinyl acetate) (PEVAc). It is a widely recognized material that is used to produce textiles, wires, cables, food packaging, toys, shoes, coating, and adhesives [1,2]. Additionally, the utilization of PEVAc has evolved into the biomedical industry, in materials such as catheters, drug delivery mechanisms, and artificial valves in heart replacement, and even potentially being used as an encapsulating material for electrical arrays in implant devices [3,4,5]. Because PEVAc has mechanical properties (tensile strength) that are superior to those of silicone elastomers, it is suitable for use in various medical instruments. According to Ahn et al. [6], silicone utilized as an implantable material has a tensile strength of 6.7 MPa, while Seok [7] reports a tensile strength of 6.2 MPs. This value of tensile strength is considered low for the purpose of use in high performance insulation material of implantable devices. In addition, PEVAc also exhibits flexibility similar to that of elastomeric materials, yet it can be processed like other thermoplastic polymers. PEVAc is also biocompatible and offers excellent optical clarity, barrier properties, and stress-crack resistance [8,9,10,11]. Because of its numerous advantages, PEVAc has been selected for various applications. However, to be used in advanced applications, such as biomedical implants, further improvement in the tensile properties, thermomechanical properties, and biostability of PEVAc are needed because these properties of PEVAc are not sufficiently high. Besides the need to improve the properties of PEVAc, a massive demand for novel materials is currently being developed due to the desire for new functions and technological advancements.

Several decades ago, scientists realized that, in comparison with a single material counterpart, mixtures of materials could produce a new form of material (composite) with excellent properties. In general, incorporating single filler/nanofillers into the structure of a polymer will create new materials called polymer composites/nanocomposites and allow improvement in the polymer’s mechanical, thermal, and barrier properties [12,13]. However, PEVAc is a copolymer comprising two types of monomers, each with a distinct property: non-polar polyethylene (PE) and polar polyvinyl acetate (PVAc). Therefore, one of the developments in the field of copolymers is the use of hybrid fillers, a combination of two or more different types of filler. Hybrid fillers were introduced in the polymer/copolymer nanocomposite field to produce an efficient material with beneficial properties that can cope with various applications. As mentioned, PEVAc is a copolymer with two different monomers and sets of properties. Hence, adding a hybrid filler has been one of the methods to improve the filler–PEVAc copolymer interaction.

Previously, research by Yuan et al. [14], Osman et al. [15], Ahmad Fauzi et al. [16], and Mohammed Fitri et al. [17] has shown that the use of PEVAc as a matrix and in hybrid fillers (two or more fillers) creates a homogenous nanocomposite with good filler dispersion. Thus, it improves the thermal stability, mechanical properties, and biostability of the resulting material compared to using a single filler. Besides the hybrid filler used, according to Osman et al. [15], Ahmad Fauzi et al. [16], and Mohammed Fitri et al. [17], the ratio between the copolymer composition and hybrid filler composition also plays a vital role in improving the performance of the copolymer. The use of OMMT and bentonite at a ratio of 2.75:0.25 (adequate ratio) improves the mechanical properties of EVA (15% vinyl acetate content) copolymer composite and reduces the thermal degradation of EVA copolymer under a physiological fluid environment, as there is an interaction between hydrophilic bentonite and hydrophilic vinyl acetate [15]. The previous study also discusses that adding organomontmorillonite (OMMT) and destabilized bentonite (hybrid filler) at a ratio of 4:1 improves the mechanical performance of PEVA (40 wt% of vinyl acetate) copolymer [16]. Mohammed Fitri et al. [17] also found that the use of hybrid or dual clay filler (surface-modified montmorillonite (S-MMT): modified bentonite (Bent-pHs)) improved the tensile performance of PEVA (40% vinyl acetate) when it was used at a ratio of 4:1. Even after three months of exposure to the physiological fluid, biostability testing demonstrated significantly more excellent tensile strength retention with less degradation. These hybrid and dual clay filler/nanofillers ensure interaction with both PEVA monomers, resulting in a homogenous composite with improved mechanical and biostability properties.

Recently, a new type of filler, dual-functionalized single-type filler, has been reported to improve the performance of copolymer. However, a limited number of studies can be found on the usage of dual-functionalized single-type filler, and none of the literature has reported using PEVAc as a matrix in conjunction with dual-functionalized single-type filler. Therefore, the following discussions are those focused on using dual-functionalized filler with different copolymers. Research by Ueda et al. [18,19] employed dual-functional polyhedral oligomeric silsesquioxane (POSS) as filler and PMMA as matrix. This dual-functional POSS has dual types of alkyl substituent, and when added to the PMMA matrix, it creates a transparent homogenous film. This dual-functional POSS simultaneously reduces the refractive index (RI) and improves the thermal and mechanical properties of the PMMA. The relationship observed between structure and function was as follows: shorter substituents significantly reduce the RI value, whereas longer alkyl chains are feasible for improving thermal stability.

In other research by Gunji et al. [20], mercaptopropyltriehoxysilane (MPTES) and vinyltriethoxysilane (VTES) double-functionalized silica was utilized as filler for rubber. Their results show that the re-dispersion of VTES-MPTES doubly functionalized silica was improved compared to that of the MPTES-silica. Their stress–strain curve was also reported to be improved compared to the MPTES-silica when both silicas were used as filler in tire rubber. This is because the functionalization of the vinyl group on the surface was adequate to achieve the re-dispersion of silica nanoparticles, and it helps to inhibit the aggregation of MPTES-functionalized silica. In addition, Ran et al. [21] reported the use of a dual-functional filler of silver-coated spherical boron nitride (Ag@s-BN), which successfully improved the thermal and electrical conductivities of silicone rubber (matrix). It was reported that the thermal conductivity was enhanced by 48.5% and 806.8% compared to the silicon rubber/s-BN and the pure silicone rubber. An increase in the electrical conductivity also led to an increase in the electromagnetic shielding effectiveness. Due to the high sphericity and smooth surface of Ag@s-BN, the mixture of Ag@s-BN-filled silicone rubber, before curing, possesses good processability and, thus, is suitable for use in potting processes. Research on green composites based on keratin with surface-functionalized cellulose nanocrystals by Song et al. [22] also found that the use of dual-functionalized nanofillers successfully improved interfacial interactions, which also leads to significantly enhanced mechanical properties and water stability of the biocomposites. Based on these studies, dual-functionalized filler can mainly improve the multiple properties of the polymer matrix by enhancing the interaction between the filler and the matrix.

Based on the information gathered above, this research employed dual-functionalized dolomite nanoparticles (DF-DNPs) as filler, with the intent of improving the mechanical performance, and thermal and biostability properties, of PEVAc copolymer. This research introduces dolomite as a filler due to its abundancy, inexpensiveness, and non-toxicity [23,24,25,26]. Dolomite, as a filler, has been reported to improve the polymer’s mechanical and thermal properties [27,28,29,30]. Dolomite’s use as a second or hybrid filler has been reported to further enhance the polymer composite’s mechanical, thermal, and physical properties [31,32,33,34]. However, there is still no research involving the dual functionalization of dolomite as a filler for PEVAc or any other copolymer. The term DF-DNPs refers to dolomite nanoparticles (DNPs) with polar (hydrophilic) and non-polar (organophilic) characteristic (P-DNPs and NP-DNPs); thus, DF-DNPs have two (dual) functionalities: (1) the non-polar DNPs can interact well with the ethylene group of the copolymer (non-polar–non-polar interaction) to allow a good stress transferring mechanism for strengthening and toughening the copolymer matrix; (2) the polar DNPs can interact well with the vinyl acetate (VA) group of the copolymer to enhance thermal stability and increase the water resistance and hydrolytic biostability of the copolymer matrix through the developed polar–polar interactions between the polar DNPs and the VA group. In this study, we show how this concept of dual functionalization is proven through data obtained from mechanical, thermal, thermomechanical, structure, and morphology studies.

## 2. Result and Discussion

This study aimed to analyze and compare the impact of raw dolomite (RD), dual-functionalized dolomite micron particles (DF-DMPs), and dual-functionalized dolomite nanoparticles (DF-DNPs) on the morphology, structure, and properties of PEVAc copolymer. In addition, this study also aimed to improve the mechanical properties, thermal properties, and biostability of the PEVAc copolymer. Hence, the structure and particle size of physically modified dolomite was first discussed. Then, the changes in structure and polarity of the dolomite before and after surface modification were studied and compared. Next, the mechanical properties of neat PEVAc, PEVAc/RD, PEVAc/DF-DMP, and PEVAc/DF-DNP samples were studied. The samples were then analyzed and compared based on their structure, morphology, thermal properties, and biostability.

### 2.1. Characterization of of Raw Dolomite (RD), Dolomite Micron Particles (DMPs) and Dolomite Nanoparticles (DNPs)

**Figure 1:**
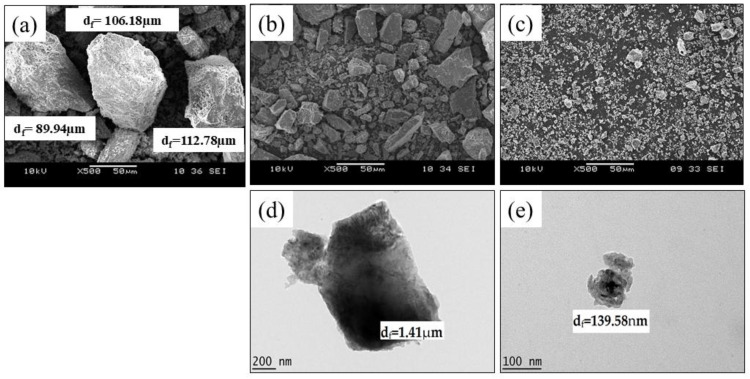
SEM micrograph of (**a**) RD, (**b**) DMPs, (**c**) DNPs; TEM micrograph of (**d**) DMPs and (**e**) DNPs.

Figure 1a shows a morphology image of raw dolomite (RD). Initially, the RD powder was supplied with a particle size of less than 150 µm, while, as shown in Figure 1a, a single/individual dolomite particle’s size can be as large as ~112.78 µm (d_f_). However, as this powder was ball-milled and ultrasonicated, a size reduction in the RD was realized, and finer dolomite particles can be spotted in the morphology image of dolomite micron particles (DMPs) and dolomite nanoparticles (DNPs) shown in Figure 1b,c.

Figure 1d,e show TEM morphology images of DMPs and DNPs, respectively. The d_f_ values for the DMPs and DNPs were ~1.41 µm and ~139.38 nm, respectively. Initially, the RD particle undergoes size reduction during the ball milling process. This decrease in the RD’s particle size could be associated with friction between the steel balls and the RD or between the RD particles, and could also be due to the contact between the RD particles and the walls of the jar. This friction could have led to the rupture of the particle’s shape edges and cleavage, thus shortening the length of the constituent particles [35,36,37,38]. Then, the size of the dolomite was further reduced when the ultrasonication method was used. The water medium allowed the travelling of sonic waves into the dolomite particles. This movement created alternating low- and high-pressure cycles. During the low-pressure cycles, microbubbles were formed which later collapsed during the high-pressure cycles in a very short time. This phenomenon is known as ultrasonic cavitation. This repetitive process caused a high local temperature, high-speed impinging liquid jets, and strong hydrodynamic shear forces. Through these effects, the dolomite particles were broken down, disintegrated, and de-agglomerated [39,40]. Thus, the particle size was significantly reduced.

The TEM morphology also revealed that DNPs (dolomite particles that underwent 50% amplitude and a repetition of 10 times) have the most significant size reduction, as the size of the dolomite particles was reduced more than the size of the DMPs (dolomite that goes through 30% amplitude and 1 times repetition). A significant decrease in particle size could be observed when higher amplitudes and more repetitions were applied. In another study, the size of vaterite also decreased with an increasing amplitude of sonication [41]. Nguyen et al. [42] also proposed that the particle size decreases with increasing frequency and amplitude. This is because the higher the amplitude, the bigger the force that the particles give to breaking themselves. Thus, a more significant size reduction can be noticed.

Figure 1e also reveals that the shapes of the DNPs were less defined (with no sharp edges) when compared to those of the DMPs, shown in Figure 1d. As mentioned earlier, a more significant impact was applied to the dolomite particles when a higher amplitude was used. This resulted in rupturing and diminished the trigonal rhombohedral (carbonate group) structure. The image of individual (de-agglomerated) DNPs particles also shows that they has a dimension of less than 100 nm (87.45 nm in width) (see Figure S1a). In contrast, a close-up image (Figure S1b) of the DNPs revealed that they possessed platy shapes with minimal thicknesses (a few nanometers only). Nevertheless, the results proved that the dolomite size can be reduced to the nano-size range by employing both ball-milling and ultrasonication methods.

**Figure 2:**
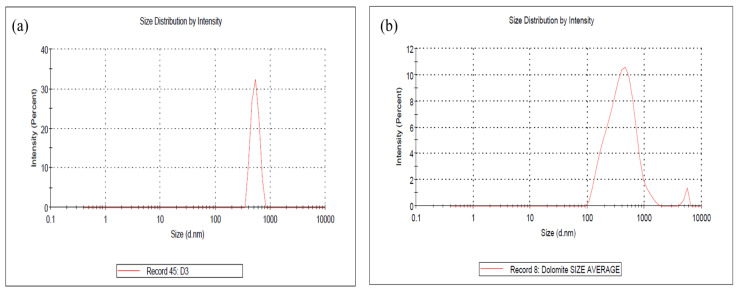
Particle size analysis of the (**a**) DMPs and (**b**) DNPs obtained through milling and tip-sonication processes.

The average particle sizes of the DMPs and DNPs were examined using a particle size analyzer (PSA). The results are presented in Figure 2a,b, showing that the average particle size of the DMPs is around 744.3 nm while that for DNPs is around 441.4 nm (in length).

It is important to highlight that this particle size analysis can only measure the diameter (length) of the nano-dolomite since it exists in a platy and irregular shape, not a uniform shape (see Figure S1a,b). The overlapping and agglomeration of several nanoparticles can occur, thus resulting in a larger scale of length measurement than the individual (separated) nanoparticles. Nevertheless, this analysis proved that the particle size of the nano-dolomite obtained through the combination of ball milling and tip-ultrasonication techniques was still in the nanometer range. Similar to other types of mineral fillers such as nanoclay, the thickness of the platy dolomite nanoparticles could be a few nanometers only (see TEM image in Figure S1a). Unfortunately, the thickness of the dolomite nanoparticles could not be measured using this particle size analyzer equipment.

Based on the SEM, TEM, and PSA, the DNPs could be defined as nanofillers with at least one dimension of less than 100 nm. The nanoparticles appeared in sheets from one to a few nanometers thick and hundreds of nanometers long. Thus, these Perlis dolomite nanoparticles could be used to produce polymer nanocomposites. The following section will detail our characterization of DMPs and DNPs as polar dolomite micron particles (P-DMPs) and polar dolomite nanoparticles (P-DNPs).

### 2.2. Characterization of NP-DMPs and NP-DNPs

**Figure 3:**
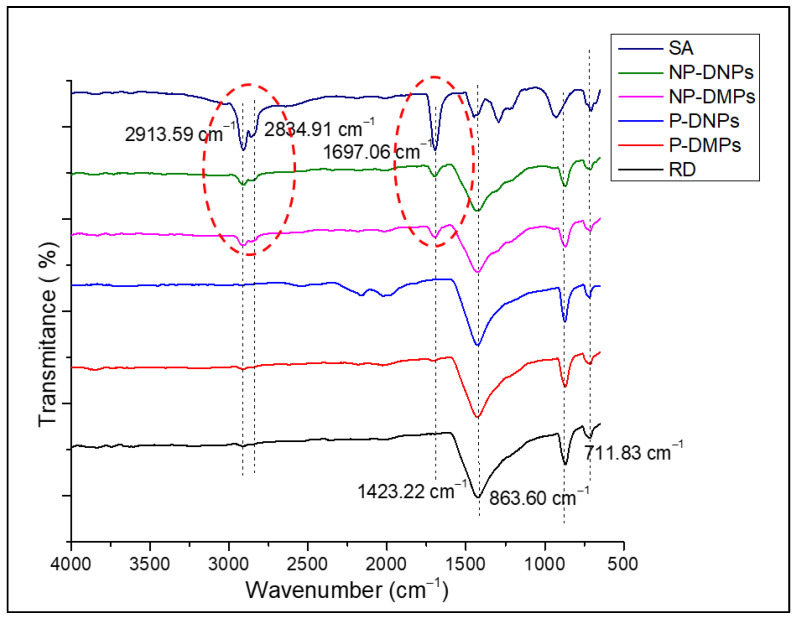
FTIR spectra of RD, P-DMPs, NP-DMPs, P-DNPs, NP-DNPs, and SA.

FT-IR analysis was performed to identify the functional groups in the P-DMPs and P-DNPs (before and after the stearic acid (SA) treatment). This ensures the change in polarity of both samples and confirms the success of the surface modification process. Figure 3 shows the FTIR spectra of the RD, P-DMPs, NP-DMPs, P-DNPs, and NP-DNPs.

The FT-IR spectra revealed the appearance of three prominent peaks of dolomite in the RD samples. The first peak was at 1423.22 cm^−1^, attributed to the asymmetric stretching vibration of the (CO_3_)^2−^ group. Other firm peaks present at 863.60 cm^−1^ and 711.83 cm^−1^ were attributed to the out-of-plane asymmetric and in-plane symmetric bending vibration mode of the O-C-O bond in the (CO_3_)^2−^ of dolomite [43,44,45]. The P-DMPs, NP-DMPs, P-DNPs, and NP-DNPs also exhibited similar peaks. Our findings agree with previously published data [44,45,46,47,48]. Ji et al. [49] and Gunasekaran et al. [50] found that the peaks at ~1420 cm^−1^, ~873 cm^−1^, and ~719 cm^−1^ are associated with dolomite.

In the SA spectrum, firm peaks were present at 2913.59 cm^−1^ and 2834.91 cm^−1^. These peaks indicated the presence of asymmetric and symmetric stretching vibrations of the aliphatic C-H group, respectively. Research by Lim et al. [51] and Chen et al. [52] also found similar results. Another peak appeared at 1697.06 cm^−1^, which indicated the presence of symmetric stretching vibrations of the C=O group from the SA.

In the NP-DNPs’ and NP-DMPs’ spectra, the prominent peaks of SA, the aliphatic group, were present at ~2900 cm^−1^ and ~2800 cm^−1^ (in red dotted circle). Another peak was also observed at ~1700 cm^−1^ (in red dotted circle), corresponding to the presence of carboxylic acid (COOH) [30]. As these typical peaks of SA were present in the NP-DNPs’ spectrum, we can confirm that the chemical surface treatment of dolomite was successful. This result agrees with other findings involving an SA treatment applied to mineral fillers, where similar peaks were reported [46,52].

The contact angle of dolomite was measured to study its wetting state before and after the chemical modification with stearic acid (SA). Table 1 shows the average contact angle of the RD, P-DMPs, and P-DNPs. The RD, P-DMPs, and P-DNPs exhibited high wettability from the water droplets, with contact angles of 53.36°, 55.12°, and 57.07°, respectively. On the other hand, the NP-DMPs and NP-DNPs showed a low wettability, with contact angles of 136.46° and 140.18°. These results further confirmed the presence of SA on the surface of the dolomite. In the FTIR analysis, the presence of the aliphatic group was realized in the NP-DMP and NP-DNP spectra. This aliphatic group provided dolomite with non-polar properties. According to Cao et al. [53], during the treatment, the aliphatic group of the SA was absorbed onto the surface of dolomite via a chemical reaction between the head part of SA and a calcium cation, thus creating a hydrophobic layer of monolayered film covering the dolomite surface. Consequently, the dolomite’s surface changed from polar to non-polar (organophilic).

### 2.3. Comparison of the Tensile Properties of Neat PEVAc, PEVAc Composite, and PEVAc Nanocomposite

In this section, P-DMPs and NP-DMPs were combined to form DF-DMPs, while P-DNPs and NP-DNPs were combined to form DF-DNPs. Both the DF-DMPs and DF-DNPs were used as fillers. In comparison, RD was also employed as a filler.

**Figure 4:**
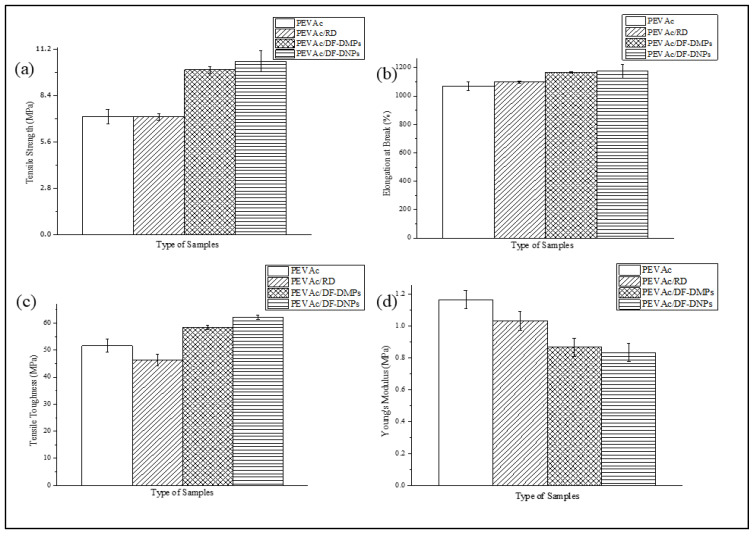
(**a**) The tensile strength, (**b**) elongation at break, (**c**) tensile toughness, and (**d**) Young’s modulus of neat PEVAc, PEVAc composite, and PEVAc nanocomposite.

Figure 4a–d represent graphs of the tensile strength, elongation at break, tensile toughness, and Young’s modulus of the neat PEVAc, PEVAc/RD, PEVAc/DF-DMPs, and PEVAc/DF-DNPs, while Table 2 summarizes the mean values of tensile strength, elongation at break, modulus elasticity, and tensile toughness of the same.

**Figure 5:**
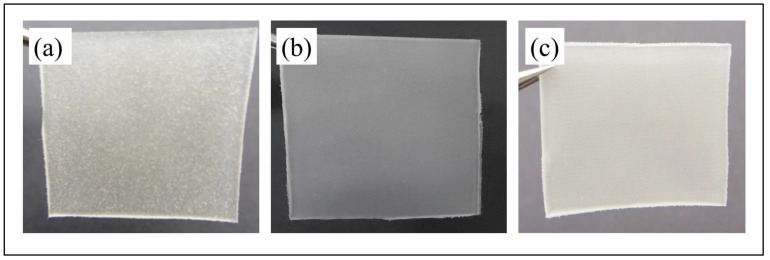
Direct image of the films; (**a**) PEVAc/RD, (**b**) PEVAc/DF-DMPs, and (**c**) PEVAc/DF-DNPs.

Initially, as shown in Figure 4a, the neat PEVAc exhibited a tensile strength of 7.14 MPa, but, with the addition of RD, the tensile strength decreased by 0.28% to 7.12 MPa. This decrement was due to the addition of the bulky particles of RD that were poorly dispersed in the PEVAc matrix, creating a non-homogeneous PEVAc composite. The direct photo of the PEVAc/RD composite shown in Figure 5a reveals the inhomogeneity of the PEVAc/RD mixture. The distribution of the agglomerated RD particles can be seen in the transparent film of the PEVAc. On the contrary, the tensile strength of the PEVAc composite with DF-DMPs as filler was found to be increased to 9.96 MPa. Compared to the neat PEVAc, the increment was reported to be 39.50%.

Interestingly, the use of DF-DNPs as filler was found to further increase the tensile strength of the PEVAc copolymer to 10.48 MPa. The increment was reported to be 46.78% compared to the neat PEVAc. This suggests that the addition of dual-functionalized DMPs and DNPs could improve the tensile strength of PEVAc. The improvement in the tensile strength of the PEVAc composite and nanocomposite was due to the well dispersed and uniformly distributed microns and nanofillers in the PEVAc matrix. Figure 5b,c also show that the films of PEVAc with DF-DMPs and DF-DNPs both have a more homogenous mixture than the PEVAc with RD.

Compared to the used of DF-DMPs as filler, the PEVAc with DF-DNPs as filler showed an increase of 5.22%. The use of nano-sized filler further increases the tensile strength to more than that obtained with the use of micron-sized filler because nano-sized filler is known to provide better filler–polymer interactions due to its larger surface area. Furthermore, small particles are more mobile and easily dispersed throughout the polymer matrix. ANOVA also revealed that the *p*-value was less than 0.05, therefore rejecting the zero hypothesis, and it can be concluded that there are statistical differences in the tensile strength value (see Table S1).

As shown in Figure 1a, RD consists of big particles with defined shapes, fewer curves, and few sharp edges, thus representing a lower-surface-area filler that can only interact poorly with the PEVAc matrix (as illustrated in Figure S2a). Meanwhile, DF-DMPs are in the submicron range size, which still results in a higher-surface-area filler than the RD. As shown in Figure 1d, even though the size of the dolomite was reduced, it still has fewer curves and few sharp edges, thus representing a lower-surface-area filler, and it interacted poorly with the PEVAc matrix (as illustrated in illustrated in Figure S2b) when compared to the interaction of PEVAc and DF-DNP filler. This was one of the reasons for the low tensile strength of the PEVAc/DF-DMPs compared to the PEVAc/DF-DNPs. On the contrary, DF-DNPs, the nano-sized filler, contained much finer particles with an irregular shape and more curves, as shown in Figure 1e. These characteristics contributed to a high-surface-area nanofiller that could more easily disperse through and interact with the PEVAc matrix (as illustrated in Figure S2c), thus improving the tensile strength.

As mentioned earlier, the tensile strengths of the PEVAc/DF-DMPs composite and PEVAc/DF-DNP nanocomposite were higher than that of the PEVAc/RD. This is because both the DF-DMP and DF-DNP fillers are more compatible with the PEVAc copolymer, which possesses hydrophobic and organophilic surface properties. PEVAc contains two different surface properties with different polarities (non-polar polyethylene chains and polar poly (vinyl acetate chains)). Thus, having dual-functional properties, both dolomites (P-DMPs and NP-DMPs or P-DNPs and NP-DNPs) can interact evenly with both monomers and, subsequently, be well dispersed and distributed throughout the PEVAc matrix. Figure S3 illustrates the interaction of dual-functionalized dolomite in the PEVAc matrix; the non-polar polyethylene (PE) phase of PEVAc copolymer interacts with non-polar DMPs or DNPs, whereas the polar poly vinylacetate (PVAc) phase interacts with polar DMPs or DNPs. The excellent interaction and interphase bonding between the PE and non-polar dolomite will allow the PE chain to stretch longer before it breaks. This non-polar dolomite can align with the PE chains, follow the strain direction, and bear the tensile force together with the PE. On the other hand, the interaction between PVAc and polar dolomite will prevent large plastic deformation of the amorphous PVAc chains and reduce the free volumes in the structure. This will ensure the sustenance of the strength and modulus of the copolymer.

The DF-DMP and DF-DNP fillers have a constant polar to non-polar ratio in the PEVAc matrix, which is 1:3 (P-DMPs or P-DNPs: NP-DMPs or NP-DNPs). This constant ratio is based on the molecule content of PEVAc (poly (vinyl acetate) phase = 25%, polyethylene phase = 75%). Previous research shows that using the correct ratio of dual or hybrid filler may improve the tensile performance of the copolymer matrix [15,16,54]. As having DF-DMPs and DF-DNPs as fillers provide better interaction of the filler with the PEVAc matrix, their use will subsequently improve the synergistic effect on the PEVAc composite/nanocomposite, in turn increasing their tensile strength. As mentioned earlier, due to the use of nano-size fillers, PEVAc/DF-DNPs has higher tensile strength than the PEVAc/DF-DMPs.

The elongation at break also showed a similar trend to the tensile strength. As shown in Figure 4b, the value decreased with the addition of RD and increased with the addition of DF-DMPs and DF-DNPs. The elongation at break of the PEVAc/RD decreased by 2.58%, changing from 1070.93% to 1098.53%. On the other hand, when compared to the neat PEVAc, the elongation at break of the PEVAc/DF-DMPs increased by 8.72%. Meanwhile, the elongation at break of the PEVAc/DF-DNPs also increased by 9.35%. However, ANOVA showed that the *p*-value was higher than 0.05; therefore, the zero hypothesis was accepted, and it was concluded that there is no statistical difference in the elongation at break value (see Table S1). This shows that the addition of RD, DF-DMPs, and DF-DNPs was able to maintain the elongation at break of the PEVAc for good resilience.

Figure 4c reveals the tensile toughness values of neat PEVAc, PEVAc/RD, PEVAc/DF-DMPs, and PEVAc/DF-DNPs. The tensile toughness was calculated based on the area under the stress–strain curve. It was revealed that the tensile toughness trend follows the trend of the composite’s tensile strength and elongation at break; the tensile toughness values of the PEVAc/DF-DMPs and PEVAc/DF-DNPs are higher than the tensile toughness of neat PEVAc. However, the tensile toughness of the PEVAc with RD decreased to the lowest tensile toughness among all samples. The tensile toughness of the PEVAc/RD decreased by 10.19%, which is 46.37 MPa from 51.63 MPa (neat PEVAc). This is due to large unmodified dolomites that hindered the polymer’s mobility chain.

Meanwhile, the increase in tensile toughness of the PEVAc/DF-DMPs and PEVAc/DF-DNPs was due to the smaller filler size, which allowed more vital filler–matrix interaction. Compared to the neat PEVAc, the toughness of the PEVAc composite with DF-DMPs increased to 58.40 MPa while that of the PEVAc nanocomposite with DF-DNPs as filler increased to 62.12 MPa. ANOVA also revealed the latter to have a *p*-value lower than 0.05; therefore, zero hypothesis was rejected, and it was concluded that there is statistical difference in the tensile toughness value (see Table S1).

As previously mentioned, the DNPs were the dolomite that underwent size reduction and de-agglomeration processes before being used as filler. Thus, when this small nano-size filler is embedded in the matrix phase, it allows the molecular motion of the copolymer chains and, at the same time, helps to transfer the stress throughout the matrix phase. These activities will enable the energy absorption mechanism during tensile deformation, which can avoid sudden break or failure. In addition, the presence of dual-functional dolomite also helps achieve a good dispersion and distribution of dolomite in the PEVAc copolymer matrix, since same-polarity interaction (PE with non-polar dolomite and PVAc with polar dolomite) might occur. The presence of dual-polarity filler makes it an “easier to disperse” filler, thus making it easier to produce a homogeneous composite. However, adding nano-sized filler further assists in creating a homogeneous PEVAc nanocomposite, thus giving PEVAc/DF-DNPs the highest tensile toughness. Meanwhile, the addition of DF-DMPs slightly lowered the tensile toughness of the PEVAc/DF-DMPs compared to that of the PEVAc/DF-DNPs. This condition causes uneven dispersion and distribution of the dolomite filler, and agglomeration of the filler in one phase of PEVAc might occur. This, in turn, would restrict the polymer chain mobility and induce sudden break or failure. As a result, the PEVAc composite with DF-DMPs cannot achieve optimum performance.

On the contrary to the tensile strength, elongation at break, and tensile toughness, as shown in Figure 4d, the Young’s modulus values for the PEVAc decreased with the addition of RD, DF-DMPs, and DF-DNPs. One of the reasons for the decrease in the Young’s modulus could be poor matrix–filler interactions which prevented an efficient stress transfer mechanism from matrix to filler. In the case of PEVA/RD, the decrement in the Young’s modulus when compared to that of neat PEVAc might be due to this reason. However, PEVAc/RD showed a more excellent Young’s modulus value, 1.03 MPa, compared to the PEVAc/DF-DMPs and PEVAc/DF-DNPs. This might be also due to RD causing the PEVAc composite to become stiffer. The stiffness of the large particles of dolomite restricted the chain mobility of the copolymer, causing the matrix to have a higher Young’s modulus [55]. The Young’s modulus of both the PEVAc/DF-DMPs and PEVAc/DF-DNPs decreased by 15.53% and 19.42%, respectively, compared to PEVAc/RD. A low Young’s modulus indicates an elastic material. The *p*-value from the ANOVA also shows that it was lower than 0.05; therefore, we rejected the zero hypothesis and concluded that there is statistical difference in the Young’s modulus value (see Table S1).

One of the reasons why the PEVAc still possessed elastic properties even though dolomite was added might be that the smaller-sized filler promotes more conformational freedom around the copolymer chain, especially in PEVAc/DF-DNPs. Thus, it can stimulate chain relaxation in the stress concentration region. It is understood that a smaller-sized filler, especially one in the nano range, is more mobile and can freely move within a matrix. In addition, NP-DNPs from DF-DNPs that interact with the PE phase might also penetrate the polymer chain and cause the PE to lose its folded chain, thus creating a more flexible chain. Therefore, with a nano-sized filler, the PEVAc nanocomposite could deform elastically. This might be why PEVAc with DF-DNPs has a lower Young’s modulus.

Overall, the results suggest a promising future for dual-functional fillers. The PEVAc/DF-DNP form demonstrates a synergistic effect, enhancing the tensile strength, elongation at break, and tensile toughness while preserving the flexibility of the PEVAc nanocomposite. This finding opens up new possibilities for improving the mechanical properties of PEVAc nanocomposites.

### 2.4. Structure and Morphology Analysis of Neat PEVAc, PEVAc Composite, and PEVAc Nanocomposite

**Figure 6:**
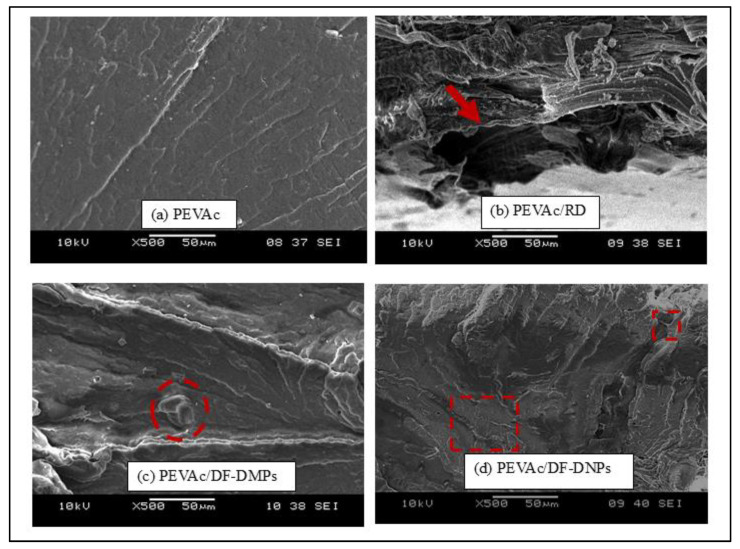
SEM micrographs of tensile-fracture surface of (**a**) neat PEVAc, (**b**) PEVAc/RD, (**c**) PEVAc/DF-DMPs, and (**d**) PEVAc/DF-DNPs.

Figure 6 shows the tensile-fracture surface morphology of neat PEVAc, PEVAc composite, and PEVAc nanocomposite. This analysis was done to study and evaluate the fracture behavior of the tested samples. Based on an SEM micrograph, neat PEVAc (Figure 6a) shows a smooth and homogenous surface; PEVAc/RD exhibits a rougher and non-homogeneous surface. As shown in Figure 6b, the PEVAc/RD micrograph also shows the presence of a large void, indicating that RD has been pulled out during the rupture of the tensile test (as shown by the arrow). The weak interfacial bonding between the RD and PEVAc caused this condition. Unmodified dolomite has a large particle size and lower surface area, thus weakening the interfacial adhesion between the dolomite filler and the PEVAc. The stress concentration between the PEVAc and RD led to poor tensile properties.

Based on Figure 6c,d, the SEM micrographs of the PEVAc/DF-DMPs and PEVAc/DF-DNPs shows a smoother and homogenous fracture surface for both compared to the fracture surface of PEVAc/RD. A homogenous PEVAc nanocomposite blend indicates a good filler dispersion. Notably, no voids were present in the PEVAc/DF-DNPs and PEVAc/DF-DMPs, suggesting that both samples formed a homogeneous composite/nanocomposite. The homogenous composite/nanocomposite film was produced because of the even and uniform filler dispersion in the PEVAc matrix. However, PEVAc/DF-DNPs show an even more homogenous surface due to the addition of nanofillers that provide a larger surface area for dolomite–PEVAc interaction. Thus, a more uniform stress-transferring mechanism between the matrix and filler subsequently increases the tensile strength and tensile toughness of the PEVAc [29,51]. The PEVAc/DF-DMPs’ micrograph also shows embedded dolomite in the PEVAc matrix (in red circle), indicating strong filler–matrix interaction. However, due to the nano size of DF-DNPs, the embedded dolomite is not recognizable. Song et al. [22] also revealed the same result: homogenous dispersion without the aggregation of filler or void was obtained using dual-functionalized filler. The micrograph of PEVAc/DF-DNPs also showed a fibrous-like structure (as shown in the red square box), indicating a strong interfacial interaction between the filler and the polymer. This can be associated with the large surface area provided by DF-DNPs, which create a large surface area for matrix–filler interactions. Small DF-DNPs could align and move together with the PEVAc chains during stretching, allowing the samples to elongate more before they break and creating the fibrous like-structure.

**Figure 7:**
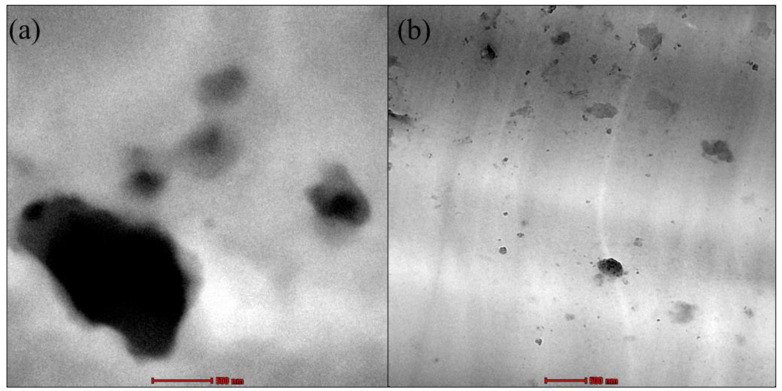
TEM micrographs of (**a**) PEVAc/RD and (**b**) PEVAc/DF-DNPs.

The dispersion and distribution of RD and DF-DNPs in the PEVAc composite were studied using TEM analysis, as shown in Figure 7a,b. The TEM image of PEVAc/RD shows the presence of large RD particles in the PEVAc; meanwhile, the TEM images of the PEVAc/DF-DNPs show better dispersion of filler in the matrix. The TEM micrograph of the PEVAc/DF-DNPs shows more evenly dispersed and distributed dolomite particles when compared to the PEVAc/RD. DF-DNPs could disperse and distribute well in the PEVAc matrix, as the two different polarities of dolomite interact with both monomers of the PEVAc matrix. In addition, filler particles with smaller sizes, mainly nanosized filler, can distribute well in the polymer since they have a larger surface area; thus, they can create a more significant area of filler–matrix interaction. This larger interface area affects the polymer’s morphology and behavior, as there is a more significant interaction between the dolomite and the polymer. In addition, it also has more edges to increase the interaction with the polymer matrix. The use of an internal mixer also helps to disperse dolomite well. The presence of a twin screw also helps to shear the agglomerated filler; thus, better dispersion of filler in the matrix can be obtained [56].

These findings agree with the tensile properties in the previous section, in which PEVAc/DF-DNPs have the highest tensile strength due to the well dispersed and distributed DF-DNP nanofiller in the PEVAc copolymer matrix. At the same time, PEVAc/RD has the weakest tensile properties due to the large dolomite hindering the mobility chain of the PEVAc.

**Figure 8:**
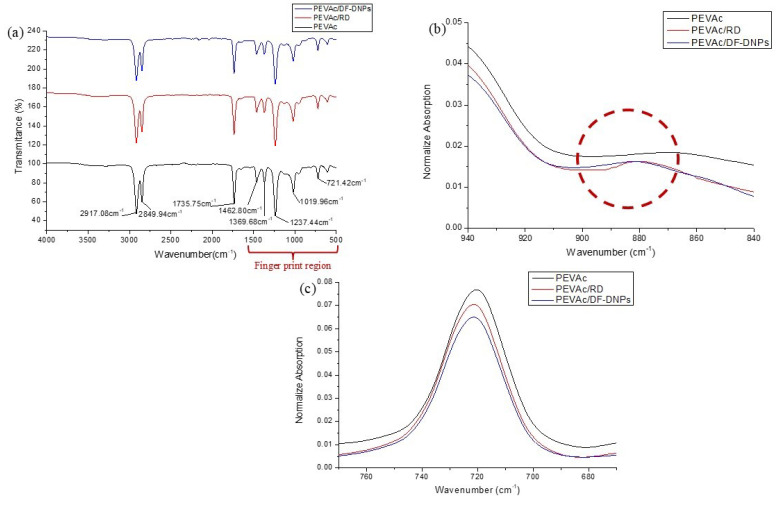
FT-IR spectra of neat PEVAc, PEVAc/RD, and PEVAc/DF-DNPs (**a**) in the ranges of 4000–500 cm^−1^, (**b**) 940–840 cm^−1^, and (**c**) 760–680 cm^−1^.

Figure 8a shows the FT-IR spectra of neat PEVAc, PEVAc/RD, and PEVAc/DF-DNPs, while Table 3 shows their band assignment. The neat PEVAc spectrum shows peaks present at 2917.08 cm^−1^, 2849.94 cm^−1^, 1462.80 cm^−1^, 1369.68 cm^−1^, and 721.42 cm^−1^, which correspond to the absorbance of the polyethylene group (PE) in the PEVAc matrix. Elanthikkal et al. [57] and Soheilmoghaddam et al. [58] reported similar findings. The peaks around 2917.08 cm^−1^ and 2849.94 cm^−1^ are attributed to the aliphatic group’s asymmetry and symmetry vibrations (-CH_2_-)_n_ of polyethylene. According to Gaston et al. [59] and Luna et al. [60], the peaks present at ~1470 cm^−1^ and ~1360 cm^−1^ are characteristic of deformation (-CH_2_-)_n_ in the main chain and deformation vibration in the plane of C-H in the CH_3_ group, respectively. The peak around ~720 cm^−1^ correlates to the long chain of CH_2_ present in the PE [59].

On the other hand, the peaks present at 1735.75 cm^−1^, 1237.44 cm^−1^, and 1019.96 cm^−1^ correlate to the absorbance of vinyl acetate in the PEVAc matrix [16,61,62]. Elanthikkal et al. [57] and Dutta et al. [63] reported that the peak present at ~1736 cm^−1^ corresponds to the carbonyl (ester) stretching vibrations of the acetate group of PEVAc. Dutta et al. [63] and Luna et al. [60] suggested that the peak present at 1220–1238 cm^−1^ correlates to the asymmetric C-O-C deformation of the acetate group and the peak at 1016–1026 cm^−1^ is attributed to the symmetric C-O-C of the acetate group. All the peaks present in neat PEVAc are also present in the FTIR spectra of PEVAc/RD and PEVAc/DF-DNPs.

Incorporating dolomite with PEVAc causes several alterations to the FTIR spectra of PEVAc/RD and PEVAc/DF-DNPs, especially in the fingerprint region between the wavenumbers from 1500 cm^−1^ to 500 cm^−1^. The FTIR spectra of PEVAc/RD and PEVAc/DF-DNPs shows the present of dolomite peak at 878.22 cm^−1^, and at 721.72 cm^−1^, respectively. This agrees with the results found by Şenol et al. [64] when dolomite was added to a polymer matrix. Hsiao et al. [65] also found two major adsorption peaks of dolomite at around 876–881 cm^−1^ and 712–730 cm^−1^. The two peaks were attributed to the O-C-O bond’s asymmetric and symmetric bending vibration modes in the (CO_3_)^2−^ of dolomite. As shown in Figure 8b,c, both peaks can be found in PEVAc/RD and PEVAc/DF-DNPs. This shows that dolomite was successfully blended in the PEVAc matrix. The peak of 721.72 cm^−1^ also indicates that adding RD and DF-DNPs causes the resulting composite’s peaks to be less intense. These changes in the peak intensity might be due to the interaction between polar dolomite and PVAc.

**Figure 9:**
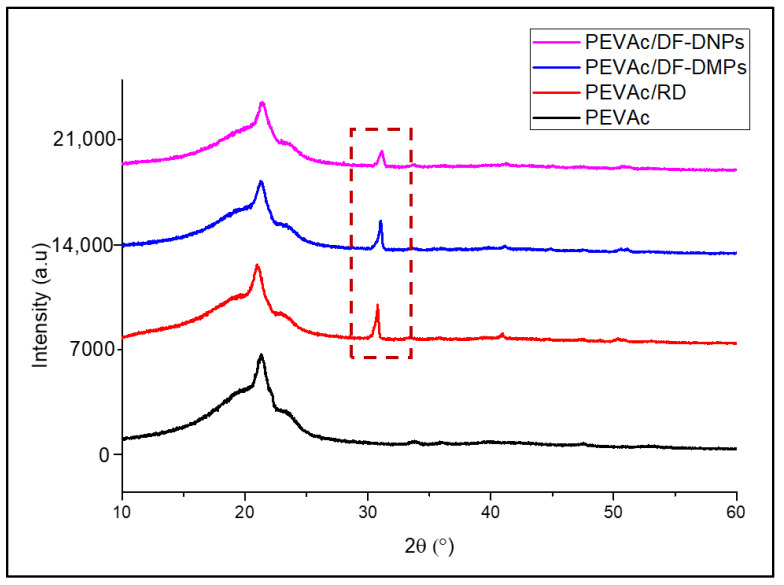
X-ray diffractogram of neat PEVAc, PEVAc/RD, PEVAc/DF-DMPs, and PEVAc/DF-DNPs in range 2θ, 10°–60°.

Figure 9 shows an XRD diffractogram of the neat PEVAc, PEVAc/RD, PEVAc/DF-DMPs, and PEVAc/DF-DNPs. Table 4 summarizes the peaks and crystallinity of the samples. The neat PEVAc’s diffractogram shows a broad peak around 2ϴ = 15–25°, which indicates the amorphous region of PEVAc. Meanwhile, the strong diffraction peak at 2ϴ = 22.40° reflects the crystalline peak of polyethylene, and the broad shoulder around 2ϴ = 24.40° is related to the crystalline peak of small ethylene blocks. This finding is in agreement with Choi and Chung’s findings [66,67]. These two peaks were also present in the PEVAc composite and PEVAc nanocomposite, and their 2ϴ values are tabulated in Table 4. All prominent peaks of PEVAc were visible in the PEVAc composite and PEVAc nanocomposite’s diffractograms. This indicates no different crystalline phases in the composite form [68].

However, one new prominent peak (in dotted box) was found in the curves of the PEVAc/RD, PEVAc/DF-DMPs, and PEVAc/DF-DNPs at 2ϴ = ~31.40°. Mohammed et al. [69] and Mantilaka et al. [70] observed that dolomite has one prominent peak around 2ϴ = ~30°. Therefore, the new peak presence at 2ϴ = 30.80°, 31.03°, and 31.17° in the curves of the PEVAc/RD, PEVAc/DF-DMPs, and PEVAc/DF-DNPs, respectively, was correlated to the presence of dolomite particles, as it was employed as filler.

Based on Table 4, PEVAc/RD has the highest degree of crystallinity due to the presence of RD. This RD is significant, and, thus, it stiffens the PEVAc composite system. The degree of crystallinity of the samples also depends on the rigidity of the sample; the higher the rigidity of the sample, the more crystalline the sample. The degrees of crystallinity of the PEVAc/DF-DMPs and PEVAc/DF-DNPs are higher than that of neat PEVAc. As mentioned earlier, the presence of dolomite stiffens the PEVAc copolymer. Thus, it increases the crystallinity of PEVAc composite and PEVAc nanocomposite. However, the PEVAc/DF-DNPs have a higher crystallinity value when compared to the PEVAc/DF-DMPs. Jing et al. [71] and Lim et al. reported that the crystallinity of a polymer composite may be influenced by the filler size and homogeneity of the filler embedded in the free volume of its polymer chains. The higher crystallinity of the PEVAc nanocomposite indicates that the nanofiller helps to increase the crystallinity of the PEVAc copolymer.

### 2.5. Thermal Properties of the Neat PEVAc, PEVAc Composite and PEVAc Nanocomposite

To determine their thermal properties, the neat PEVAc, PEVAc composite, and PEVAc nanocomposite were analyzed with TGA, DSC, and DMTA for their thermal stability, thermal behavior, and thermomechanical properties, respectively.

**Figure 10:**
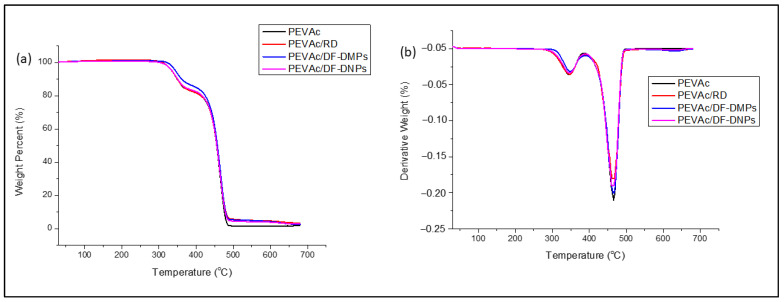
(**a**) TGA thermograms for neat PEVAc, PEVAc composite, and PEVAc nanocomposite and (**b**) DTG curves for neat PEVAc, PEVAc composite, and PEVAc nanocomposite.

The thermal stability of the materials was analyzed and compared based on their T_dmax_ and weight loss (%). Figure 10a,b illustrate the TGA and DTG curves of the neat PEVAc, PEVAc/RD, PEVAc/DF-DMPs, and PEVAc/DF-DNPs, respectively. Table 5 summarizes the T_dmax_ (°C) and weight loss (%) in the PEVAc decomposition process’s first and second steps.

Generally, neat PEVAc has two steps of decomposition. The first decomposition step was from around 300 °C to 400 °C and was associated with double bond formation (C=C) and the elimination of acetic acid [72,73,74,75]. The second decomposition step was around 420–500 °C and was related to the oxidation and volatilization of hydrocarbon due to the decomposition of residual hydrocarbon along the main chain PEVAc copolymer [72,73,74,75]. The neat PEVAc, PEVAc/RD, PEVAc/DF-DMPs, and PEVAc/DF-DNPs exhibit similar thermal decomposition behaviors.

Table 5 shows that, in the first step, the T_dmax1_ of neat PEVAc occurred at 345.9 °C, whereas that of the PEVAc/RD occurred at 344.27 °C, lower than that of neat PEVAc. This might be due to the presence of RD (unmodified dolomite). The unmodified dolomite has a large size and strong polar properties, which cause poor PVAc–dolomite interaction and poor thermal stability. However, the T_dmax1_ values of the PEVAc/DF-DMPs and PEVAc/DF-DNPs were 347.11 °C and 346.36 °C, respectively, and these values were higher than that of neat PEVAc. This composite and nanocomposite have polar dolomite that interacts with the polar PVAc of the PEVAc matrix; thus, higher energy is required to break the PVAc chain. The increase in T_dmax1_ might also be related to the present polar dolomite that can provide a barrier effect to the diffusion of the volatile thermal decomposition product of the matrix [76]. In addition, the presence of dolomite can restrict the polymer chain and provide excellent heat insulation. PEVAc/DF-DMPs have higher T_dmax1_ than the PEVAc/DF-DNPs. The presence of nano-sized filler might also influence the reduction in thermal stability of the PEVAc nanocomposite. It was also found that the nano-sized calcite has lower thermal stability than the common calcite due to the nano effect, which is also expected to happen in nanodolomite [77].

In the second step, the thermal degradation process of the PEVAc/RD happens faster than in the neat PEVAc. The T_dmax2_ of the neat PEVAc was 465.76 °C, while the T_dmax2_ of the PEVAc/RD was 463.26 °C, as shown in Table 5. As previously mentioned, RD has poor compatibility with the PE component, leading to a reduction in the thermal degradation temperature. The T_dmax2_ values of the PEVAc/DF-DMPs and PEVAc/DF-DNPs show a slight decrease compared to neat PEVAc, consistent with the findings of Nguyen et al. [78]. This decline may be attributed to the presence of SA, which may give a catalytic effect during the thermal degradation process. However, the T_dmax2_ of PEVAc/DF-DNPs is lower than the T_dmax2_ of PEVAc/DF-DMPs, due to the nano effect mentioned earlier.

In the first step, the PEVAc/RD, PEVAc/DF-DMP, and PEVAc/DF-DNP samples show lower weight loss than neat PEVAc. However, both the PEVAc/DF-DMPs and PEVAc/DF-DNPs show lower weight loss when compared to the PEVAc/RD. This indicates that dolomite and PVAc interactions improve thermal stability. The second step of weight loss displays that the PEVAc/RD, PEVAc/DF-DMPs, and PEVAc/DF-DNPs have higher weight loss than the neat PEVAc. However, among the PEVAc nanocomposites, PEVAc/DF-DNPs have the lowest weight loss, indicating that using DF-DNPs improved the thermal stability of the PEVAc copolymer.

**Figure 11:**
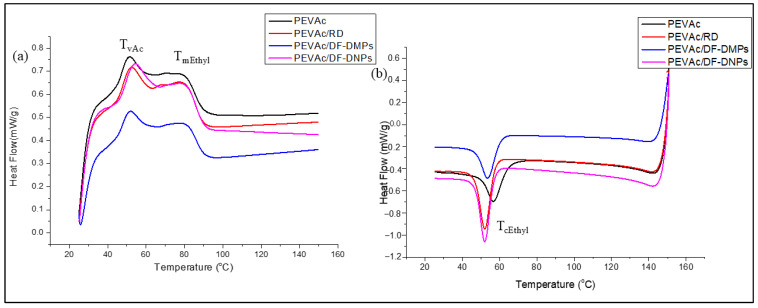
DSC (**a**) heating curves and (**b**) cooling curves of neat PEVAc, PEVAC composite, and PEVAc nanocomposite.

DSC analysis was conducted to investigate the thermal transition and correlation between the thermal behavior and molecular structure of the neat PEVAc, PEVAc/RD, PEVAc/DF-DMP, and PEVAc/DF-DNP samples. According to Almeida et al. [79], the vinyl acetate (PVAc) content in PEVAc influences its glass transition temperature, polymer crystallinity, melting point, and flexibility. A higher PVAc content results in lower crystallinity because the overall crystallinity of the copolymer is determined by the thickness of the lamellae, mainly comprising ethylene units. The PVAc phase is amorphous, unlike the ethylene phase. Therefore, the melting temperature of PEVAc copolymers is a function of the average sequence length of its crystallizable ethylene units. The melting and crystallization behavior of the neat PEVAc and selected PEVAc composites and nanocomposites were evaluated through DSC heating and cooling curves, as shown in Figure 11a,b, respectively. Table 6 represents a summary of the softening PVAc temperature (T_VAc_), melting temperature of ethylene-phase PEVAc (T_mEthyl_), enthalpy of fusion of the ethylene melting endotherms (ΔH_mEthyl_), cooling temperature of the ethylene phase (T_cEthyl_), enthalpy of crystallization of the ethylene units (ΔH_cEthyl_), and degree of crystallinity (Xc%) of the neat PEVAc, PEVAc/RD, PEVAc/DF-DMPs, and PEVAc/DF-DNPs.

Figure 11a shows two endotherm peaks in the neat PEVAc, PEVAc/RD, PEVAc/DF-DMP, and PEVAc/DF-DNP heating scans. They were labeled as T_VAc_ and T_mEthyl_. The T_VAc_ endotherm peaks at ~50 °C of the neat PEVAc, PEVAc composite, and nanocomposite was due to the softening temperature of PVAc in the PEVAc matrix. In contrast, the T_mEthyl_ endotherm peak at ~80 °C was due to the melting point of the PEVAc copolymer. Other findings were reported to have the same results [80,81,82].

Initially, the T_VAc_ of the neat PEVAc was 51.07 °C and its T_mEthyl_ was 79.84 °C. However, the addition of dolomite as filler influenced both the T_VAc_ and T_mEthyl_. The T_mEthyl_ values of the PEVAc/RD, PEVAc/DF-DMPs, and PEVAc/DF-DNPs increase to 51.97 °C, 51.57 °C, and 53.93 °C, respectively. This is because adding RD, DF-DMPs, and DF-DNPs enables them to interact with the PVAc phase. Thus, to break this interaction, a higher temperature was required. In addition, the PEVAc/DF-DNPs have a higher T_VAc_ than the PEVAc/DF-DMPs. Although dual-functionalized dolomite was employed in both the composite and nanocomposite, the nano-sized dolomite in the PEVAc/DF-DNPs allows stronger interfacial adhesion between the polar dolomite nanofiller and PEVAc, as nanosized filler provide a large surface area for matrix–filler interaction.

In addition, as referred to in Table 6, the T_mEthyl_ values of the PEVAc/RD, PEVAc/DF-DMPs, and PEVAc/DF-DNPs are higher than that of neat PEVAc, indicating that the presence of dolomite delayed the melting process of PEVAc. However, the T_mEthyl_ of PEVAc/RD is lower than that of the PEVAc/DF-DMPs and PEVAc/DF-DNPs. This could be attributed to either no or poor interaction between the PEVAc matrix and RD, which possess polar characteristics. In contrast, introducing DF-DMPs and DF-DNPs as fillers may result in a stronger interaction between the PEVAc matrix and the filler, thus requiring higher temperatures to break the bond between them. Including DF-DNPs successfully increases the T_VAc_, T_mEthyl_, and maximum thermal degradation values of PEVAc copolymer. This is because of the suitable interaction of DF-DNPs with both monomers from the PEVAc copolymer.

Crystallinity refers to the level of structural organization in a solid. In the case of PEVAc, the polyethylene (PE) phase, with its irregular lamellar structure, provides the crystallinity properties. In contrast, unlike the ethylene phase, the polyvinyl acetate (PVAc) phase possesses an amorphous structure. As in Table 6, the crystallinity of PEVAc/RD is the highest when compared to neat PEVAc, PEVAc/DF-DMPs, and PEVAc/DF-DNPs. As mentioned earlier regarding the XRD analysis, RD is a large, unmodified dolomite with rigid properties, and the rigidity and stiffness of a material influence its crystallinity. Thus, having RD as filler creates a rigid and stiff PEVAc composite. The higher the stiffness, the higher the crystallinity. This is the reason for there being a higher PEVAc/RD crystallinity despite the low tensile strength.

**Figure 12:**
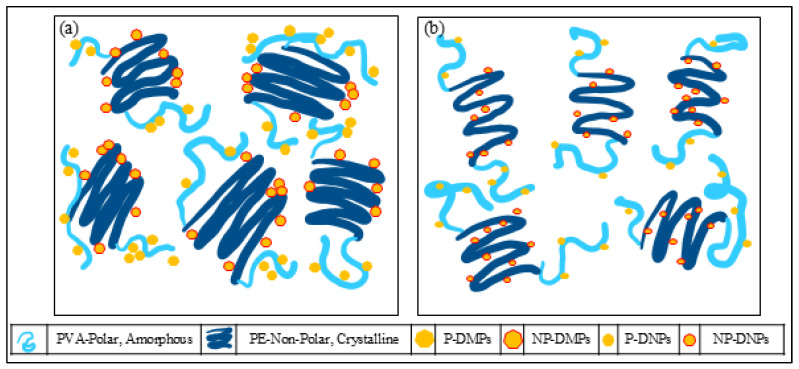
Illustration of (**a**) DF-DMPs and (**b**) DF-DNPs distribution in PEVAc matrix.

However, similar to the XRD results, the PEVAc/DF-DMPs have lower crystallinity than the PEVAc/DF-DNPs even though the size of the DF-DMPs is larger than that of DF-DNPs. This is because the DF-DMPs are of submicron size; thus, they not big enough to stiffen the PEVAc copolymer as RD did. However, their size might restrict the mobility of the PEVAc chain, as DF- DMPs still interact with both monomers of PEVAc. Figure 12a,b illustrate the distribution of DF-DMPs and DF-DNPs in the PEVAc matrix. According to Sabzi et al. [83], the decrease in crystallinity at low nanoparticle concentrations might also be due to the dominant influence of nanoparticle confinement. On the other hand, Lasmi et al. [84] stated that the reduction in the crystallinity of well compounded nanocomposites might result from the strong interaction developed between the compounds coexisting in the system, and, as well, the increase in compatibility between the polymer matrix and filler also restricts the free movement of polymer chains, which could obstruct the spherulite development stage of the ethylene fraction in the copolymer.

Based on the results shown in Figure 11b, the cooling curve shows only one prominent crystallization exotherm peak, which was around ~50 °C for the neat PEVAc, PEVAc/RD, PEVAc/DF-DMPs, and PEVAc/DF-DNPs. This cooling peak was labeled with T_cEthyl_. Generally, the crystallization of the polymer matrix can be affected by the nucleation effect of the nanoparticle (nanofiller). The nucleation process involves atomic or molecular rearrangements of reactant phase particles, resulting in the formation of a cluster of the product phase that is capable of growing irreversibly into a larger size [85]. PEVAc/RD, PEVAc/DF-DMPs, and PEVAc/DF-DNPs have lower T_cEthyl_ values than the T_cEthyl_ of neat PEVAc. This is obviously due to the presence of dolomite. The ΔH_cEthyl_ values of PEVAc/RD and PEVAc/DF-DNPs are higher than that of neat PEVAc. However, PEVAc/DF-DMPs have a lower ΔH_cEthyl_ value. PEVAc/DF-DNPs have the highest ΔH_cEthyl_, indicating that nano-size dolomite acts as a nucleating agent during the crystallization of PEVAc/DF-DNPs. Based on this result, DF-DNPs can act as driving forces for the crystallization of the PE phase. This is due to the compatibility of the nanofiller and the crystalline PE phase. According to Ahmad Fauzi et al. [16], well dispersed nanofillers could interact better with the EVA matrix and serve more efficiently as nucleating agents.

**Figure 13:**
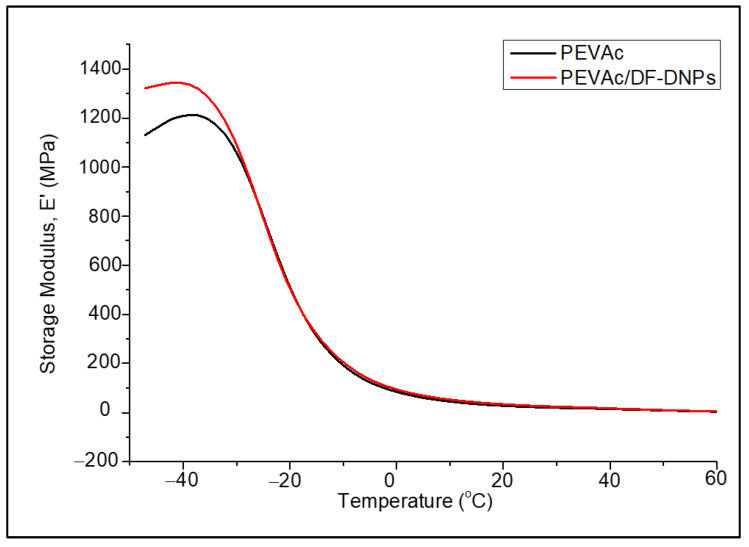
The E’ of neat PEVAc and PEVAc/DF-DNPs.

DMTA was done to analyze the morphological and thermomechanical performances of the neat PEVAc and PEVAc/DF-DNPs. This test was used to study their stiffness and mechanical damping, and the results were reported as modulus and tan ẟ. The dynamic mechanical properties of a polymer blend depend on its crystallinity, molecular structure, and extent of crosslinking [86]. The viscoelasticity properties were evaluated based on three physical properties, which were storage modulus (E’), loss modulus (E”), and tan ẟ as functioned to temperature. E’ measures the energy which is stored in the sample and which will be released after mechanical stress. At the same time, E” is the measure of the energy dissipated as heat when the material turns viscous. Tan ẟ is defined by E”/E’. Tan ẟ represents the elastic response due to the stored elastic energy in the materials, which, in turn, stands for the material’s viscous response when subjected to deformation or viscous dissipation. Tan ẟ is also known as the damping factor. In this chapter, the discussion was focused on E’ and tan ẟ. Figure 13 illustrates a graph of the E’ of neat PEVAc and PEVAc/DF-DNPs.

At the temperature range from −50 °C to −25 °C (glassy region), the E’ of PEVAc/DF-DNPs is higher than that of the neat PEVAc. The increase in the E’ of PEVAc/DF-DNPs was due to the rigidity of the DF-DNPs, which imparted their stiffness behavior to the filled PEVAc nanocomposite [87]. The addition of DF-DNPs allows the non-polar dolomite nanoparticles to interact with the non-polar PE phase, and the polar dolomite nanoparticles to interact with the PVAc phases of PEVAc. This interaction will enable DF-DNPs to have strong interfacial adhesion with the PEVAc and allow the filler to be dispersed and distributed throughout the PEVAc matrix. Thus, it stiffens the PEVAc matrix. Zubkiewicx et al. [88] also share a similar view, in which the high E’ can be associated with the enhancement in composite stiffness due to strong interfacial and intertubular interactions between the matrix and filler.

The E’ decreases with further temperature increases for the neat PEVAc and PEVAc nanocomposites, and this was attributed to the softening of the polymer matrix at higher temperatures. This decrement was also accompanied by a maximum damping capacity peak (tan ẟ max) in the tan ẟ thermogram from around −30 °C to 30 °C. In this temperature range, the E’ of the PEVAc nanocomposite is higher than that of the neat PEVAc.

**Figure 14:**
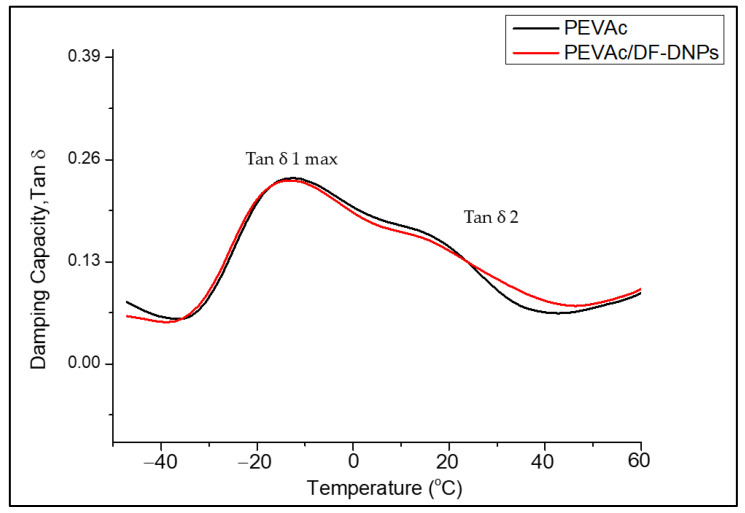
The damping capacity (tan ẟ) of neat PEVAc and PEVAc/DF-DNPs.

Figure 14 illustrates a graph of the tan ẟ of the neat PEVAc and PEVAc/DF-DNPs. As mentioned earlier, tan ẟ measures the damping behavior and is expressed as the ratio of the E” to the E’ [89]. According to Stark et al. [90], a weak peak at a temperature of −120 °C is related to the relaxation peak. However, a peak was not visible in the neat PEVAc and PEVAc/DF-DNPs curve as the DMTA reached −50 °C.

As shown in Figure 14, at temperatures from −20 °C to 30 °C (T_g1_), a relaxation temperature with a pronounced maximum damping capacity peak (tan ẟ max) was noticed. Wang and Deng [91] reported that this is related to the glass transition of the amorphous phase in PEVAc, consisting of rigid amorphous PE and amorphous PVAc segments. The curves of neat PEVAc and PEVAc/DF-DNPs were revealed to exhibit the same trend. Still, the tan ẟ max of PEVAc/DF-DNPs is slightly shifted toward lower temperatures (as in Table 7) than that of neat PEVAc, indicating that it is more flexible or the same as neat PEVAc. Even though there was strong interfacial adhesion between the polymer and filler, having nano-sized particles distributed through the matrix did not restrict the movement of the polymer chain. The decrement of the tan ẟ’s max might also be due to the energy dissipation process becoming slower with the addition of DF-DNPs. This indicates a reduction in the crystallinity of the system, which enhanced the molecular motion in the amorphous phase [86].

A shoulder peak (tan ẟ 2) was noticed at temperatures from 10 °C to 30 °C (T_g2_) for the neat PEVAc and PEVAc/DF-DNP curves. However, adding DF-DNPs alters the damping capacity shoulder of PEVAc by making it less intense. This shoulder is related to the rigid amorphous fraction represented by the portion of the amorphous phase in the PEVAc crystallites. This shows that interaction between polar dolomite nanoparticles and polar PVAc occurs, leading to the decrement of tan ẟ 2 from 18.77 °C to 17.43 °C [82,92].

### 2.6. Biostability Analysis

Biostability assessment was also done on the PEVAc/DF-DNPs and was compared with that of the neat PEVAc, PEVAc/RD, and PEVAc/DF-DMPs. The samples were immersed in a physiological salt solution called phosphate-buffered saline (PBS) at 37 °C for 3 months during the biostability study. This environment is referred to as in vitro, and it simulates human body fluids, making it an ideal testing environment for medical devices. Mohammed Fitri et al. [17] claimed that, after being exposed to an in vitro environment, the copolymer may degrade through an oxidation mechanism in which the interactions between oxygen molecules and the ions (H+ and OH) in the PBS solution produce more free radicals to accelerate the oxidation process. This radical can react with the polar molecules of the PEVAc and be transferred to other parts of the copolymer chains. In addition, the biostability of the PEVAc copolymer may also be reduced through the physical degradation process, in which it may undergo water-induced swelling, which would affect its dimensional stability, glass transition temperature (T_g_), and mechanical properties. The biostability assessment in this study featured a comparison of the water resistance, tensile properties, and tensile fractured surface’s degradation morphology.

A water resistance test was done to study the hydration characteristics of neat PEVAc, PEVAc/RD, PEVAc/DF-DMPs, and PEVAc/DF-DNPs. The water resistance of polymer is affected by several factors, such as its structure, its composition, and the addition of nanofiller. Most polymers naturally have a propensity to absorb water. The observed increase in mass was assumed to be a result of water permeability or absorption in the polymer matrix [93]. The increase in mass may be influenced by the degree of dispersion of the filler, weaknesses at the polymer–filler interface, disrupted molecular packing, or an increase in the size of the free volume elements in the polymer. This factor affects the liquid water permeability of the polymer nanocomposite, which can increase water transmission through the polymer [94,95,96,97,98].

**Figure 15:**
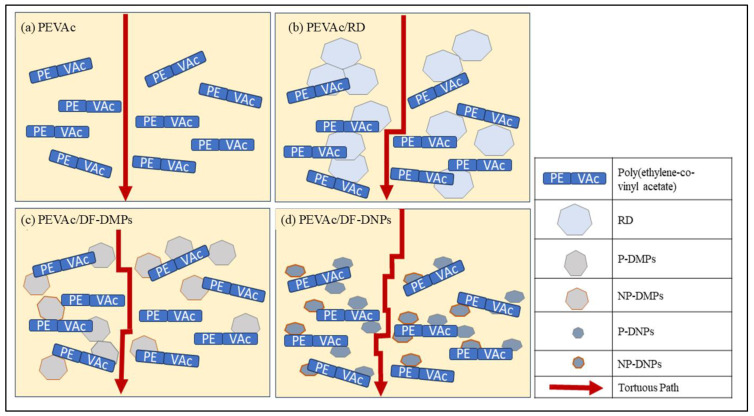
Illustration of the tortuous pathway of (**a**) PEVAc, (**b**) PEVAc/RD, (**c**) PEVAc/DF-DMPs, and (**d**) PEVAc/DF-DNPs.

In this study, the sample weight was recorded every two weeks for three months and these are presented in Table 8. It was discovered that every sample increased in weight upon exposure to the in vitro environment. However, the PEVAc/DF-DNPs showed the most minor increase in weight percentage when compared to the neat PEVAc, PEVAc/RD, and PEVAc/DF-DMPs. After 12 weeks, the weight of the PEVAc/DF-DNPs was increased by 2.63%; meanwhile, the neat PEVAc, PEVAc/RD, and PEVAc/DF-DMPs gained weight by 6.10%, 5.01%, and 4.62%, respectively. This might be due to the ability of DF-DNPs to retain the water permeability in the PEVAc matrix, as they was well dispersed and distributed due to being able to interact with both PEVAc monomers. Figure 15 illustrates the tortuous pathway of the PEVAc, PEVAc/RD, PEVAc/DF-DMPs, and PEVAc/DF-DNPs. Better dispersion in the filler structure introduced a more tortuous path for water molecule diffusion than poor filler dispersion (as in Figure 15d), thus decreasing or restricting their permeability towards the polymer molecular chain [99]. The polar PVAc phase of the PEVAc matrix contributes to the hydrophilicity characteristics of the PEVAc chain. Thus, having polar dolomite in DF-DMPs and DF-DNPs prevents water permeability towards the PVAc chains, since polar dolomite interacts with PVAc. In addition, nano-sized fillers also provide a large surface area for dolomite PEVAc interaction, thus making it more difficult for water molecules to travel toward the PVAc. This explains why PEVAc/DF-DNPs have the most minor increment in weight after 3 months of immersion in PBS solution.

The PEVAc/DF-DMPs showed a higher increment in mass than the PEVAc/DF-DNPs, although both had dual-functionalized dolomite filler. This might be due to the micron size of the DF-DMP filler, which reduces the bond strength between the filler and matrix since it has a lower surface area, thus allowing the water permeability to be pushed towards the PEVAc chain. As in Figure 15c, the PEVAc/DF-DMPs have a less tortuous path than the PEVAc/DF-DNPs. The PEVAc/RD showed the highest percentage increase in weight due to the presence of RD. Although RD has polar properties due to being unmodified, the interaction between it and PEVAc is weak, thus allowing water permeability towards the PEVAc chain, especially the PVAc phase. In addition, it also may lead to a less tortuous path for water molecules, which enables the water to permeate towards the polymer molecular chain (as in Figure 15b) and degrade the PEVAc.

**Figure 16:**
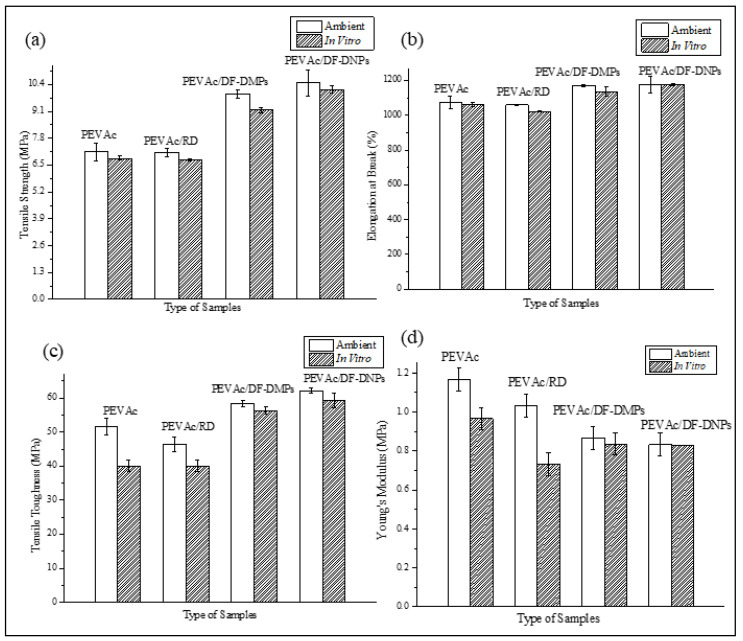
The (**a**) tensile strength, (**b**) elongation at break. (**c**) tensile toughness, and (**d**) Young’s modulus of neat PEVAc, PEVAc/RD, PEVAc DF-DMPs, and PEVAc/DF-DNPs before and after immersion in in vitro environment.

The primary objective of this study was to conduct a tensile test to evaluate the biostability of various composite films, including neat PEVAc, PEVAc/RD, PEVAc/DF-DMPs, and PEVAc/DF-DNPs. This was achieved by comparing the tensile properties of the samples before and after exposure to an in vitro environment. Figure 16a–d present the tensile strength, elongation at break, tensile toughness, and Young’s modulus of the neat PEVAc, PEVAc/RD, PEVAc/DF-DMPs, and PEVAc/DF-DNPs before and after exposure. Table 9 provides a comparison of the average data of the tensile strength, elongation at break, tensile toughness, Young’s modulus, and reduction percentage of the samples before and after immersion in the in vitro conditions.

Based on the obtained results, all samples showed a reduction in tensile strength after exposure to the in vitro physiological fluid. The tensile strength of the neat PEVAc, PEVAc/RD, PEVAc/DF-DMPs, and PEVAc/DF-DNPs was reduced by 4.34%, 5.06%, 6.93%, and 3.05% after being exposed to the in vitro condition, respectively. The reduction in tensile strength was expected, as water molecules can occupy the hydrogen bonding sites and hinder the secondary bonding between copolymer chains. In addition, the elongation of break of the neat PEVAc, PEVAc/RD, PEVAc/DF-DMPs, and PEVAc/DF-DNPs also shows a reduction of 1.35%, 0.42%, 2.97%, and 0.13%, respectively. The reduction in the tensile toughness of the neat PEVAc, PEVAc/RD, PEVAc/DF-DMPs, and PEVAc/DF-DNPs was 6.00%, 13.67%, 3.67%, and 4.43%, respectively. The Young’s modulus of the neat PEVAc, PEVAc/RD, PEVAc/DF-DMPs, and PEVAc/DF-DNPs was reduced by 34.18%, 29.13%, 4.60%, and 3.61% after exposure for three months to the in vitro condition. This might be because the absorbed water acts as a plasticizer for the polymer, thus reducing the rigidity of the polymer.

Based on the tensile results, it was noticeable that the PEVAc/DF-DNPs have the most significant water resistance after exposure to the in vitro condition. Besides having the smallest percentage of reduction, the PEVAc/DF-DNPs maintain their highest tensile strength, elongation at break, and tensile toughness even after exposure to the in vitro environment. This is because the addition of DF-DNPs reduces the degradation process. The good interfacial adhesion between the PEVAc matrix and DF-DNP nanofillers might minimize degradation. Good interfacial adhesion occurs as DF-DNPs interact with both phases in PEVAc, leading to a good dispersion and distribution of the polymer nanocomposite system. The excellent filler dispersion in the polymer matrix also reduces the permeability of the water molecules and oxidative agents into the copolymer chain’s structure, creating a tortuous path for the entrance of these permeants. Thus, it will resist the attack of hydrolytic and oxidative agents on the copolymer chains. Mohamed Fitri et al. [17] also suggest that the PVAc phase (more susceptible to degradation) of a copolymer that contains easy-to-hydrolyze non-carbon atoms can be protected by the presence of polar nanofiller in the dual-functionalized nanofiller through the developed strong polar–polar bonding between the polar nanofiller and the PVA molecular chains. This is because the rigid structure of the polar nanofiller can reduce the hydrolytic activity of the more vulnerable bonds of the PVA chains via steric hindrance. In this research, due to the presence of P-DNPs in DF-DNPs, the interaction between the PVAc and P-DNPs occurs. Thus, this interaction reduces the hydrolytic activity of the more vulnerable bonds of the PVAc chains via steric hindrance, and the rigidity of the P-DNPs further reduces the permeability of water. In addition, the dolomite itself may also restrict fluid passage into the PVA chains.

It was also revealed that the percentage reductions in the tensile strength, elongation at break, and Young’s modulus of PEVAc/DF- DMPs were higher than those of PEVAc/DF-DNPs. As mentioned, a larger-size filler might create a less tortuous path (as in Figure 15c) and a weak filler–matrix interaction, thus allowing water permeability and leading to the faster occurrence of degradation. In agreement with the water permeability results, the PEVAc/RD shows the highest percentages of reduction in the tensile strength, elongation at break, modulus of elasticity, and tensile toughness when compared to the PEVAc/DF-DMPs and PEVAc/DF-DNPs. The sizeable dolomite size creates a less tortuous path. In addition, weak RD–PEVAc interactions also allow the water’s entrance toward the polymer chain. This leads to a faster degradation process via hydrolysis, oxidation, and the physical process.

**Figure 17:**
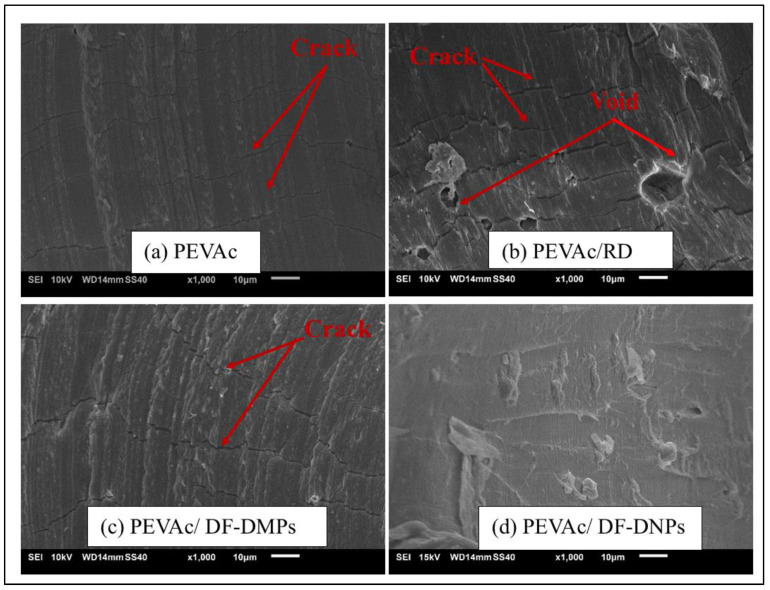
SEM images of fracture surface of (**a**) neat PEVAc, (**b**) PEVAc/RD, (**c**) PEVAc/DF-DMPs, and (**d**) PEVAc/DF-DNPs after immersion in in vitro environment.

SEM analysis was done on the tensile fracture surface of the neat PEVAc, PEVAc/RD, PEVAc/DF-DMPs, and PEVAc/DF-DNPs by comparing the tensile-fracture surface morphology of the samples after exposure to the in vitro condition. The SEM images of all samples are shown in Figure 17.

Based on Figure 17, signs of degradation can be noticed, as the tensile fractures of neat PEVAc, PEVAc/RD, and PEVAc/DF-DMPs show bumpier and cracked surfaces after exposure to the in vitro environment. The degradation of the PEVAc composite can be associated with its water permeability, for which the more significant the permeability, the greater the surface degradation that can be seen. The PEVAc/RD shows the roughest surface with voids compared to the neat PEVAc and the PEVAc/D-DMPs. This agrees with the water resistance test, as the increase in the mass (%) of the PEVAc/RD was the highest. RD, as a filler (polar and unmodified), attracts greater water permeation, as the RD and PVAc do not have strong bonds. Thus, more severe degradation occurred through hydrolysis, causing the break-up of bonds and chains.

On the other hand, the SEM analysis of PEVAc with DF-DNPs shows a smoother, less bumpy surface compared to the other different composites. The dual nanofiller plays a crucial role in reducing the host copolymer’s permeability, creating a more tortuous path for the passage of the simulated body fluid [17]. The nano-sized filler further reduces degradation by improving the interaction between the filler and matrix, leading to a more biostable copolymeric material [17]. These results suggest that the presence of DF-DNPs significantly enhances the biostability of PEVAc copolymers.

## 3. Materials and Methods

### 3.1. Materials

Poly (ethylene-co-vinyl acetate) (PEVAc) copolymer with 25% vinyl acetate composition was utilized as the matrix material. It was in bead form with hardness 28 (shore D, ASTM D 2240), melt index of 19 g/10 min (190 °C/2.16 kg), and 0.948 g/mL density. Its melt temperature (T_m_) is ~110–120 °C, and its transition temperature (T_g_) is around 46 °C (Vicat, ASTM D 1525). Its linear formula is (CH_2_CH_2_)m[CH_2_CH(OCOCH_3_)]n. In addition, dolomite was used as a filler/nanofiller; stearic acid (SA) was utilized to surface-modify the dolomite, while isopropyl alcohol was employed to dissolve SA. Dolomite in pulverized form was supplied by Perlis Dolomite Industries (PDI) with an average particle size of less than 150 µm, in a white-beige color, and with a chemical formula of CaMg(CO_3_)_2_. SA (C_18_H_36_O_2_) used has a molecular weight of 284.498 g/mol, and it appeared in platy shape and white color solid, with a melting range of 66–69 °C and density of 0.847 g/cm^3^. Finally, the isopropyl alcohol (2-propanol) used has a chemical formula of C_3_H_8_O, a boiling point of 82.5 °C, and a density of 786 kg/m^3^.

### 3.2. Preparations of Dual-Functionalized Dolomite Filler

Dual-functionalized dolomite nanoparticles (DF-DNPs) were prepared by combining polar dolomite nanoparticles (P-DNPs) and non-polar dolomite nanoparticles (NP-DNPs). P-DNPs are dolomites that have been physically modified to a nano size. At the same time, NP-DNPs are dolomites that have been physically modified to nano size and chemically modified to have organophilic or non-polar properties. For comparison, dual-functionalized dolomite micron particles (DF-DMPs) were also prepared. DF-DMPs were prepared by combining polar dolomite micron particles (P-DMPs) and non-polar dolomite micron particles (NP-DMPs). P-DMPs are dolomites that have been physically modified to have a submicron size. At the same time, NP-DMPs are dolomites that have been physically modified to have a submicron size and chemically modified to have organophilic or non-polar properties.

#### 3.2.1. Preparation of P-DMPs and P-DNPs via Ball Milling and Ultrasonication

At first, the Fritsch Pulverisette planetary mill (Bayern, Gemany) was employed to grind the raw dolomite (RD) with 50 pieces of grinding balls of 15 mm diameter and a mass of 13.78 g each. The speed used was 500 rpm with a duration of 6 h. According to Nik Adik et al. [100], a longer duration (higher than 6 h) causes a plateau effect on the dolomite particle size. Guzzo et al. [101] and Chen et al. [35] found that the size of particles increased as the speed increased. Chen et al. [35] reported that higher speed may cause the particles to agglomerate.

**Figure 18:**
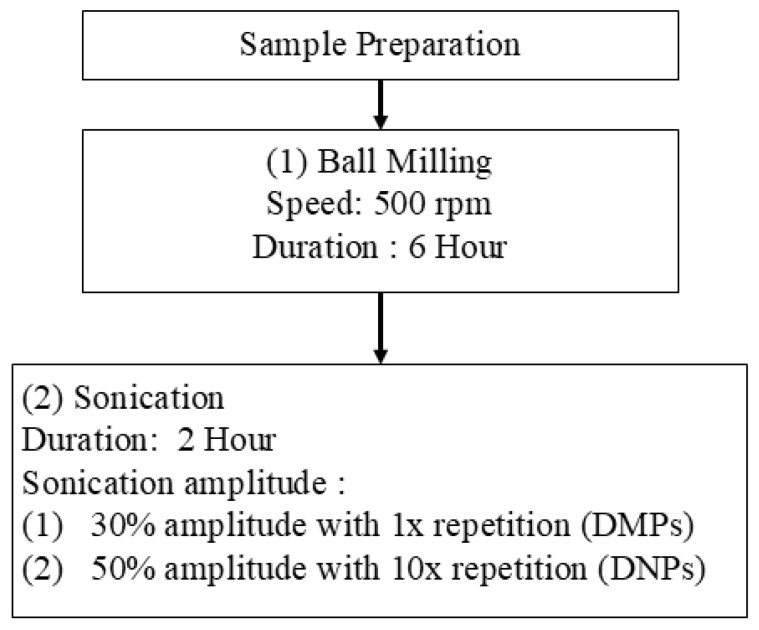
The overview of the physical modification process of dolomite with ball-milling and ultrasonication.

Secondly, to further reduce the size of the dolomite, 20 g of the ball-milled dolomite was dispersed in 100 mL of distilled water. Then, the dolomite was sonicated for 2 h with 10 s pulse on, 10 s pulse at 30% amplitude, and 1× repetition for DMPs and at 50% amplitude with 10× repetition for DNPs. Branson Digital Ultrasonic Disrupter/Homogenizer, model 450 D (Queensland, Australia) was used for this sonication process. Then, the sample was centrifuged for 10 min at 4000 rpm using a Rotofix 32 A benchtop centrifuge machine (Tuttlingen, Germany), and the samples were dried in an oven at 80 °C for 24 h before being ground and sieved. P-DMPs and P-DNPs were used in dual-functionalized dolomite filler for the PEVAc copolymer matrix. Figure 18 illustrates the overview of the physical modification process of dolomite with ball-milling and sonication parameters together with the sample acronyms.

#### 3.2.2. Preparation of NP-DMPs and NP-DNPs

DMPs and DNPs were surface-modified with SA. This surface modification technique changed the hydrophilic surface of dolomite into an organophilic (hydrophobic) surface. Initially, 10 g of DMPs was weighed before adding 100 mL of distilled water. DMP suspension was stirred for 15 min at 50 °C. Then, 0.16 g of SA was dissolved in 10 mL of isopropyl alcohol at 50 °C. The dissolved SA was added to the dolomite suspension and stirred for 3 h using a homogenizer. The suspension was centrifuged at 4000 rpm for 10 min and dried in the oven at 80 °C for 24 h. Finally, the sample was sieved for further use in testing and characterization. The same surface modification procedures were applied to the DNP sample. The surface-modified DMPs were called NP-DMPs, while the surface-modified DNPs were called NP-DNPs.

P-DMPs and NP-DMPs were combined to form DF-DMPs. P-DNPs and NP-DNPs were combined to form DF-DNPs. Both DF-DMPs and DF-DNPs were used as fillers. RD was also employed as filler for comparison purposes.

### 3.3. Preparations of Neat PEVAc, PEVAc Composite and PEVAc Nanocomposites

Table 10 summarizes the formulation of the PEVAc composite and PEVAc nanocomposites, with their respective acronyms. PEVAc composite represents the PEVAc/RD and PEVAc/DF-DMPs, while PEVAc nanocomposite represents PEVAc/DF-DNPs. PEVAc composites and nanocomposites were prepared with 3 wt% of dolomite loading, regardless of the type of dolomite, by using an internal mixer (IM). In addition, the ratio of non-polar/polar dolomite in composite and nanocomposite with dual-functionalized filler was kept constant at 3:1. From previous research, the ratio of hybrid or dual-polarity filler that follows the copolymer’s polar and non-polar monomer ratio gave the best mechanical performance [15,16,17]. In this study, the ratio of PE:PVAc = 3:1; thus, the ratio of NP-DNPs:P-DNPs used was also 3:1.

Initially, the pre-weight PEVAc pellets were first discharged into the mixing chamber and allowed to melt for about 3 min. Then, dolomite filler was added and the sample was compounded for 10 min to achieve a homogenous composite. The mixer was operated at 160 °C and 36 rpm speed. Next, the compounded samples were collected and compressed into a sheet that was 1 mm thick and 225 cm^2^ in area in a mold by a compression molding machine, model GT-7014-H30C by GOTECH Co. (Taichung City, Taiwan). The samples were preheated at 160 °C for 5 min, then pressed for 4 min and cooled for 7 min. Finally, the sample was cut according to the characterization and testing requirements.

### 3.4. Characterization

#### 3.4.1. Scanning Electron Microscopy (SEM)

**Figure 19:**
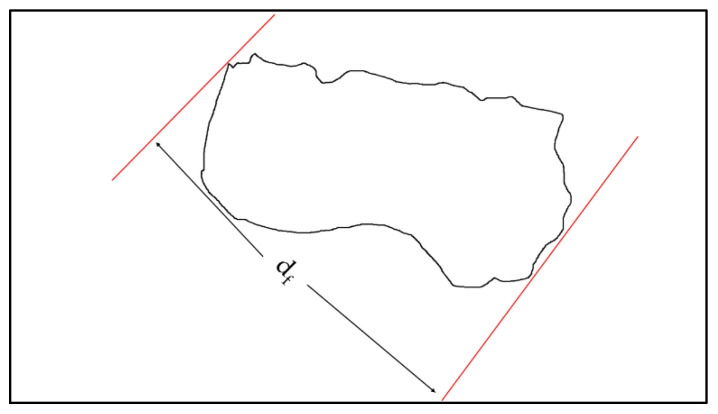
The illustration of Feret diameter (d_f_) of a particle.

SEM analysis was done to characterize the morphology of dolomite, mainly to observe the size reduction in dolomite particles (before and after being milled and sonicated). Meanwhile, the neat PEVAc, PEVAc composite, and PEVAc nanocomposite samples were analyzed based on their fractured surface upon tensile failure. SEM analysis was done using a SEM, model SEM-JEOL JSM-6460LA (JEOL, Tokyo, Japan) with 10 kV of voltage. The size of dolomite particles was calculated based on Feret diameter (d_f_) as shown in Figure 19, and the d_f_ was obtained from imageJ. Feret diameter refers to the longest distance between any two points along the selected boundary (see Figure 19). This measurement is more suitable for irregularly shaped particles like dolomite [102,103].

#### 3.4.2. Transmission Electron Microscopy (TEM)

TEM analysis was done on dolomite filler to compare the dolomite size reduction (DMPs and DNPs). The states of filler dispersion, morphology, and inner structure of the PEVAc/RD and PEVAc/DF-DNP samples were also investigated by TEM analysis. TEM analysis was carried out by using JEOL JEM2010 Electron Microscope (Tokyo, Japan). The sample was cut to 300 nm thickness using a glass cutter on a Leica Utracut Ultramicrotome (UCT) instrument at T_g_ temperature = −80 °C and then maintained using liquid nitrogen. Then, the sample was picked up to the 200-mesh Cu grid using a 2.5 M sucrose solution. Lastly, the sample was air-dried under a covered petri dish before viewing.

#### 3.4.3. Particle Size Analyzer (PSA)

Before the PSA analysis, 0.15 mg of dolomite (DMPs or DNPs) was dispersed in 2 mL of solvent (toluene). This procedure separated large aggregates and agglomerates in the sedimentation, allowing the suspension to stand for 1–2 h. Subsequently, the particle size distribution was measured with a Malvern Instruments Zetasizer Nano ZSP using NIBS technology and dynamic light scattering (Malvern Panalytical Ltd., Malvern, UK).

#### 3.4.4. X-Ray Diffractometer (XRD)

A Bruker D2 phaser benchtop X-ray diffractometer (Bruker Corporation, Billerica, MA, USA) was used to characterize the changes in and crystallinity of neat PEVAc, PEVAc composite, and PEVAc nanocomposite. The sample was analyzed from 10° to 60° with a step size of 0.022 and a time per step of 19.2 s. This X-ray diffractometer operated at 30 kV using Cu Ka α rays (λ = 0.15406). The crystallinity or peak-to-noise ratio of the samples was calculated using Equation (1):

(1) $$XRD\;(\%)=\frac{Ic}{\left(Ic+IA\right)}\times 100\%$$

where Ic is the area of the crystalline peaks of the sample, which was obtained by calculating the area under the crystalline peaks, and (Ic + IA) is the total area under all the sample peaks.

#### 3.4.5. Fourier Transform-Infrared Spectroscopy (FTIR)

FT-IR analysis was done to identify the functional groups and the differences in the FT-IR spectra of the RD and surface-modified dolomite. Perkin Elmer RXI FT-IR spectrophotometer (Waltham, MA, USA) was employed. The FT-IR analysis was done via the ATR method in the 4000–650 cm^−1^ range. The spectra were recorded with 16 scans and a resolution of 4 cm^−1^. The FT-IR analysis was also done on the neat PEVAc, PEVAc composites, and PEVAc nanocomposites.

#### 3.4.6. Contact Angle Analysis

Contact angle analysis was performed to determine the surface characteristics and wettability of the dolomite (before and after surface was treated with SA). Dolomite powder was placed onto a thin layer of plasticine on top of a glass slide, and then flattened to get a smooth surface. Next, distilled water was dropped onto the dolomite surface using a syringe. The droplet image was captured by a direct phone camera with a microlens attached. The contact angle was analyzed and calculated using ImageJ version 1.53 software with a drop analysis plug-in. Each sample had three angles measured, and the average value was calculated.

#### 3.4.7. Thermogravimetry Analysis (TGA)

TGA analysis was performed to study the thermal stability of the neat PEVAc, PEVAc composite, and PEVAc nanocomposites using TGA Pyris Diamond Perkin Elmer 6 equipment. The test was conducted by using a constant heating rate of 10 °C/min from ambient temperature to 900 °C under a nitrogen atmosphere for raw and surface-treated dolomite; meanwhile, for the neat PEVAc, PEVAc composite, and PEVAc nanocomposites, it was until 700 °C. The thermal stability of the materials was compared through their percentage of weight loss and maximum temperature for degradation (T_dmax_), which were obtained through the TGA and derivative thermogravimetric (DTG) curves.

#### 3.4.8. Tensile Analysis

The tensile properties of neat PEVAc, PEVAc composites, and PEVAc nanocomposites were evaluated and compared. The samples were cut according to ASTM D-638-M-5 to obtain dumbbell-shaped samples. The tensile test was performed using Instron machine model-5582 (Instron^®^, Norwood, MA, USA) with a 50 mm/min crosshead speed. The mean values of tensile strength, elongation at break, tensile toughness, and Young’s modulus were recorded.

#### 3.4.9. Differential Scanning Calorimetry Analysis (DSC)

DSC analysis was carried out using DSC heating and cooling curves to investigate the thermal transition and melting behavior of the neat PEVAc, PEVAc composites, and PEVAc nanocomposites. The test was done by using a DSC Q10 analyzer. The sample was analyzed from room temperature until 150 °C with a heating rate of 10 °C/min under a nitrogen atmosphere. In the cooling process, the sample was analyzed from 150 °C to room temperature with a cooling rate of 10 °C/min. To compare the thermal behavior between materials, their melting temperature (T_mEthyl_), enthalpy of fusion (ΔH_mEthyl_), crystallization temperature (T_cEthyl_), and enthalpy of crystallization (ΔH_cEthyl_) were observed. The degree of crystallinity (Xc) of neat PEVAc, PEVAc composite, and PEVAc nanocomposites was calculated with Equation (2).

(2) $$Xc\left(\%\right)=\frac{∆H_{mEthyl}}{∆H_{o}}\times 100\%$$

ΔH_methyl_ is the melting enthalpy of samples, and is calculated from the second endotherm peak, while ΔH_o_ is the theoretical melting enthalpy of 100% PE crystalline polymer, which is 277.1 J/g [104].

#### 3.4.10. Dynamic Mechanical Thermal Analysis (DMTA)

DMTA analysis was conducted to analyze the thermomechanical properties of the neat PEVAc, PEVAc composite, and PEVAc nanocomposites. The test was performed using Perkin Elmer Instrument at 0.1% strain in tension mode with a frequency of 1 Hz. The measurement started at −40 °C and ended at 120 °C using the heating rate of 2 °C/min. The sample was tested in the tension mode with a length of 50 mm, width of 7 mm, and thickness of 3 mm.

#### 3.4.11. Biostability Analysis

To perform biostability analysis, specific samples were chosen and placed in a phosphate-buffered saline (PBS) solution with a pH of approximately 7.4. The solution was maintained at a temperature of 37 °C, similar to the temperature of the human body. The sample was immersed in the solution for 3 months using a water bath method. The test was done using the standard test method for water absorption of plastic (ASTM D 570). This experiment was conducted to simulate the fluid conditions of the human body. The samples were weighed every 2 weeks for a water resistance test, and, after 3 months, the samples were subjected to the tensile test and their fracture surface was analyzed. Both tensile testing and SEM analysis involved the same procedure, shown in Section 3.4.1 and Section 3.4.8, respectively.

#### 3.4.12. Water Resistance Test

A water resistance test was done to study the hydration characteristics of the neat PEVAc, PEVAc composite, and PEVAc nanocomposites. This study was related to the in vitro biostability performance. This test was done by weighing the immersed sample every 2 weeks to analyze the hydrolytic permeability behavior, while the percentage of weight change was calculated based on Equation (3).

(3) $$Increase\;in\;mass\;\left(\%\right)=\frac{(Final\;mass-Initial\;mass)\times 100}{Initial\;mass}$$

## 4. Conclusions

In this research, dual-functionalized DF-DNPs were prepared and used as a nanofiller in a poly (ethylene-co-vinyl acetate) copolymer nanocomposite system. DF-DNPs are nano-sized dolomite particles with polar and non-polar properties. They could quickly disperse in the copolymeric matrix due to having both a small particle size and dual polarity, thus serving as an efficient reinforcing filler. PEVAc/DF-DNPs significantly improve the composite’s tensile properties compared to the neat PEVAc, PEVAc/RD, and PEVAc/DF-DMPs. This is due to the excellent dispersion and distribution of dolomite in the PEVAc matrix, as the nano-sized filler has a higher surface area, thus allowing it to better interact with the PEVAc matrix. TEM images revealed that the PEVAc/DF-DNPs nanocomposite contains filler that is dispersed and distributed well throughout the matrix. This shows that the DF-DNPs interact well with the PEVAc matrix. Combining both P-DNPs and NP-DNPs to form dual-functionalized dolomite (DF-DNPs) produced an “easier to disperse” nanofiller to, in turn, create a homogeneous nanocomposite. Thus, this DF-DNP nanofiller could improve the tensile performance of the PEVAc copolymer. The thermal analysis results indicate that the DF-DNPs can improve the overall maximum thermal degradation (T_dmax_) and melting temperature (T_mEthyl_) of the PEVAc copolymer. The thermomechanical analysis also reveals that the PEVAc/DF-DNPs have flexibility similar to that of the neat PEVAc despite having dolomite as a filler. Biostability assessment also showed that using DF-DNPs as a nanofiller caused the PEVAc copolymer to achieve the best water resistance, tensile properties, and retention in surface degradation.

## Figures and Tables

**Table 1:** The average contact angle of RD, P-DMPs, P-DNPs, NP-DMPs and NP-DNPs.

Samples	Average Angle (°)
RD	53.36 ± 0.15
P-DMP	55.12 ± 0.45
P-DNPs	57.07 ± 0.25
NP-DMPs	136.46 ± 0.32
NP-DNPs	140.18 ± 1.72

**Table 2:** The summary of the mean values of the tensile strength, elongation at break, modulus elasticity, and tensile toughness of neat PEVAc, PEVAc composite, and PEVAc nanocomposite with different types and sizes of dolomite.

Sample	Tensile Strength (MPa)	Elongation at Break (%)	Tensile Toughness (MPa)	Modulus of Elasticity (MPa)
PEVAc	7.14 ± 0.43	1070.93 ± 31.12	51.63 ± 2.43	1.17 ± 0.058
PEVAc/RD	7.12 ± 0.20	1098.53 ± 7.74	46.37 ± 2.13	1.03 ± 0.058
PEVAc/DF-DMPs	9.96 ± 0.21	1168.90 ± 5.25	58.40 ± 0.82	0.87 ± 0.06
PEVAc/DF-DNPs	10.48 ± 0.63	1175.73 ± 49.54	62.12 ± 0.83	0.83 ± 0.06

**Table 3:** The band assignments of FTIR spectra of neat PEVAc, PEVAc composites, and PEVAc nanocomposites.

Samples and Wavenumber (cm^−1^)			Bands
PEVAc	PEVAc/RD	PEVAc/DF-DNPs	
2917.08	2917.09	2916.99	Asymmetric vibration of aliphatic (-CH_2_-)n
2849.94	2849.97	2849.82	Symmetric vibration of aliphatic (-CH_2_-)n
1735.75	1736.16	1735.81	-CO carbonyl stretching vibration of esters
1462.80	1462.81	1462.52	(-CH_2_-)n deformation
-	1434.26	1434.44	Asymmetric stretching vibration of the (CO_3_)^2−^ group.
1369.68	1369.73	1369.61	(-CH_2_-)n wagging
1237.44	1237.48	1237.28	The asymmetric vibration of C-O-C bond
1019.96	1020.07	1019.80	C-O-C in plane stretching
-	879.79	880.35	Asymmetric bending vibration mode of the O-C-O bond in the (CO_3_)^2−^
721.42	721.44	721.38	CH bending (out-of-plane)

**Table 4:** The peak and crystallinity of neat PEVAc, PEVAc composite, and PEVAc nanocomposite.

Samples	Crystallinity	Amorphous	Peak (2ϴ)		
			1	2	3
PEVAc	17.82	82.18	21.36	23.51	-
PEVAc/RD	26.06	73.94	21.03	23.43	30.80
PEVAc/DF-DMPs	17.96	82.04	21.35	23.60	31.03
PEVAc/DF-DNPs	18.07	81.93	21.38	23.76	31.17

**Table 5:** Comparison of T_dmax_ and weight loss (%) of neat PEVAc, PEVAc composite, and PEVAc nanocomposite in the first and second steps of the PEVAc decomposition process.

Samples	T_dmax 1_ (°C)	Weight Loss (%)	T_dmax 2_ (°C)	Weight Loss (%)
PEVAc (IM)	345.90	19.08	465.76	77.6
PEVAc/RD	344.27	18.84	463.26	79.2
PEVAc/DF-DMPs	347.11	15.20	465.32	80.65
PEVAc/DF-DNPs	346.36	17.31	463.89	79.16

**Table 6:** Summary of DSC heating and cooling curve of neat PEVA, PEVAc composites, and PEVAc nanocomposite.

Samples	T_VAc_ (°C)	T_mEthyl_ (°C)	ΔH_mEthyl_ (J/g)	Xc (%)	T_cEthyl_ (°C)	ΔH_cEthyl_ (J/g)
PEVAc	51.07	79.84	8.23	2.98	56.96	17.51
PEVAc/RD	51.97	79.85	9.88	3.56	52.19	23.61
PEVAc/DF-DMPs	51.57	80.29	6.43	2.32	53.89	13.75
PEVAc/DF-DNPs	53.93	80.58	7.92	2.86	52.12	25.15

**Table 7:** The DMTA damping capacity of PEVAc, PEVAc/DNPs, PEVAc/NP-DNPs, and PEVAc/DF-DNPs.

Samples	Tg1 (°C) (Tan ẟ Max)	Tg2 (°C) (Tan ẟ 2)
PEVAc	−13.52	18.77
PEVAc/DF-DNPs	−13.45	17.43

**Table 8:** Increment percentage of neat PEVAc, PEVAc/RD, PEVAc/DF-DMPs, and PEVAc/DF-DNPs after immersion in PBS solution (37 °C, 3 months).

Weeks	Increment of Weight in Samples Upon Hydrolytic Agent (PBS Solution) (%)			
	PEVAc	PEVAc/RD	PEVAc/DF-DMPs	PEVAc/DF-DNPs
0	0	0	0	0
2	1.22	1.43	0.97	0.48
4	2.20	2.39	1.70	0.72
6	3.66	2.63	2.68	1.20
8	4.63	3.34	3.65	2.15
10	4.87	4.53	4.38	2.39
12	6.10	5.01	4.62	2.63

**Table 9:** The summarization of the average value of tensile strength elongation at break, tensile toughness, and Young’s modulus of neat PEVAc, PEVAc/RD, PEVAc/DF-DMPs, and PEVAc/DF-DNPs before and after immersion in in vitro environment.

Sample	Condition	Tensile Strength (MPa)	Elongation at Break (%)	Tensile Toughness (MPa)	Young’s Modulus (MPa)
PEVAc	A	7.14 ± 0.43	1075.73 ± 22.60	51.63 ± 2.42	1.17 ± 0.06
	I	6.83 ± 0.10	1061.2 ± 10.81	48.54 ± 4.53	0.77 ± 0.06
	Reduction (%)	4.34	1.35	6.00	34.18
PEVAc/RD	A	7.12 ± 0.20	1025.97 ± 1.40	46.38 ± 2.13	1.03 ± 0.058
	I	6.76 ± 0.05	1021.63 ± 2.58	40.04 ± 1.78	0.73 ± 0.06
	Reduction (%)	5.06	0.42	13.67	29.13
PEVAc/DF-DMP	A	9.96 ± 0.21	1168.90 ± 5.25	58.40 ± 0.82	0.87 ± 0.06
	I	9.27 ± 0.12	1134.23 ± 26.45	56.26 ± 1.07	0.83 ± 0.06
	Reduction (%)	6.93	2.97	3.66	4.60
PEVAc/DF-DNP	A	10.48 ± 0.63	1175.73 ± 49.54	62.12 ± 0.83	0.83 ± 0.06
	I	10.16 ± 0.16	1174.10 ± 5.23	59.37 ± 2.15	0.80 ± 0.0000135
	Reduction (%)	3.05	0.13	4.43	3.61

A: ambient, I: in vitro.

**Table 10:** The formulation and acronyms of neat PEVAc, PEVAc composite, and PEVAc nanocomposites.

Type of Sample	Matrix (PEVAc) (wt %)	Filler (Dolomite) (wt%)			Acronym
PEVAc	100	-			
		Type of Filler	Weight (wt%)		PEVAc
PEVAc/RD	97	RD	3%		PEVAc/RD
PEVAc/(DF-DMPs) (P-DMPs + NP-DMPs)	97	DF-DMPs (P-DMPs + NP-DMPs) (P-DMPs:NP-DMPs = 1:3)	3%		PEVAc/DF-DMPs
			P-DMPs	0.75	
			NP-DMPs	2.25	
PEVAc/(DF-DNPs) (P-DNPs + NP-DNPs)	97	DF-DNPs (P-DNPs + NP-DNPs) (P-DNPs:NP-DNPs = 1:3)	3%		PEVAc/DF-DNPs
			P-DNPs	0.75	
			NP-DNPs	2.25	

## Data Availability

There are no linked research dataset for this submission. Data will be made available on request.

## Supplementary Materials

The following supporting information can be downloaded at: https://www.mdpi.com/article/10.3390/ijms252312519/s1.
