# Supplementary material for: Structure and Properties of Poly(Ethylene-co-vinyl Acetate) Nanocomposites with Dual-Functionalized Dolomite Nanoparticles
Source: Int J Mol Sci. 2024 Nov 21;25(23):12519. doi: 10.3390/ijms252312519 (PMC11641816; doi:10.3390/ijms252312519)
Supplement: Supplementary file 1 [file ijms-25-12519-s001.zip › ijms-3252669-supplementary.pdf]

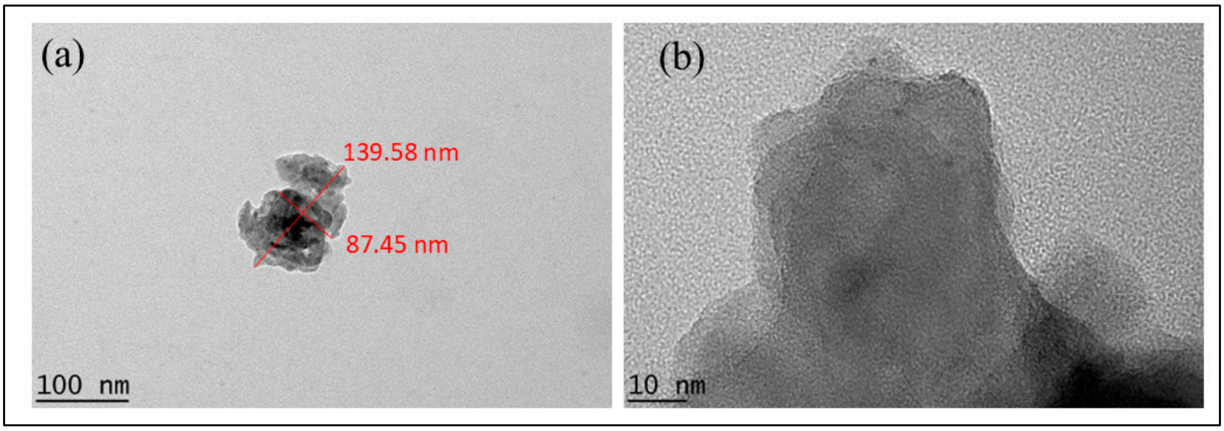

Figure S1. TEM micrographs of (a) DNPs with the measured length and width and (b) platy/sheet-like particles of DNPs with a very small thickness (less than 100 nm).

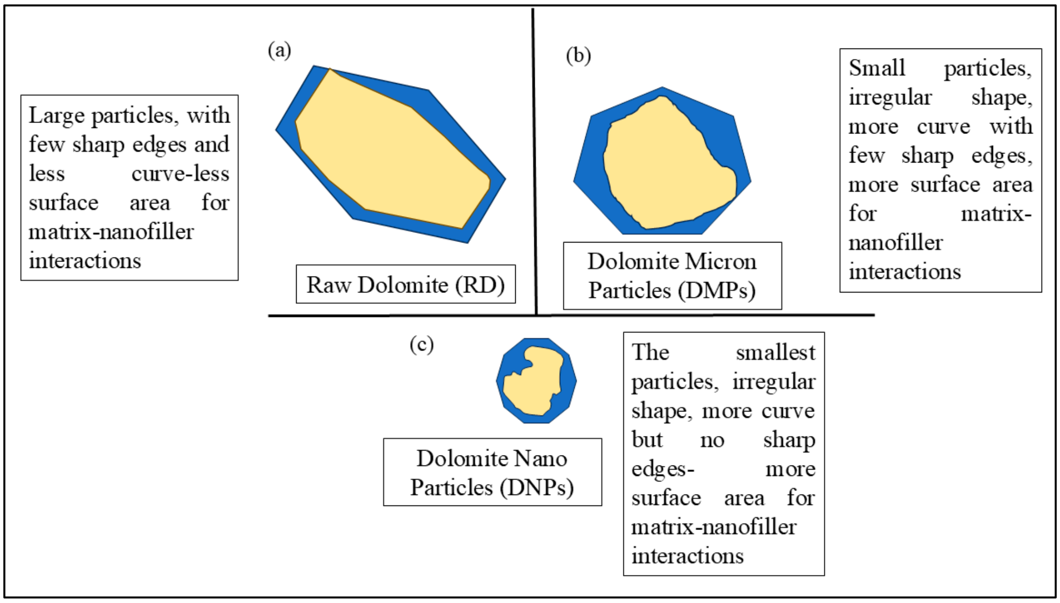

Figure S2. Illustration of (a) RD, (b) DMPs), and (c) DNPs structure in PEVAc matrix.

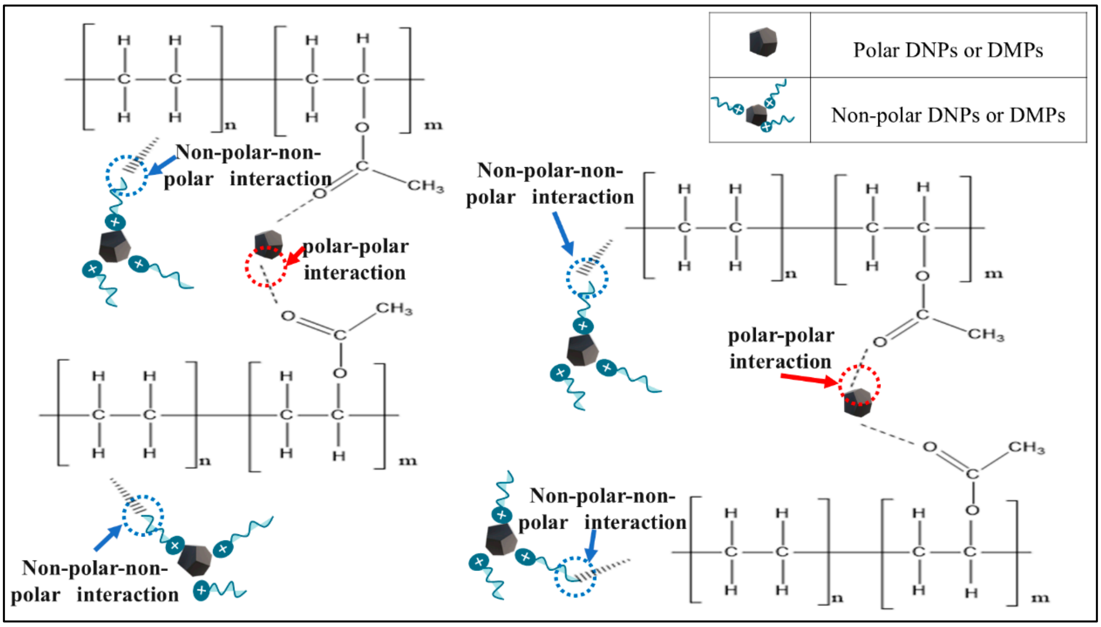

Figure S3. The illustration of the interaction between DMPs or DNPs and PEVAc copolymer.

Table S1. The single factor ANOVA result of tensile strength (TS), elongation at break (EAB), tensile toughness (TT) and Young's modulus (YM)

Sample	*Source of Variation*	*SS*	*df*	*MS*	*F*	*P-value*	*F crit*	
TS	Between Groups	29.00442	3	9.668141	57.8712	9.1E-06	4.066181	significant
EAB	Between Groups	24225.2	3	8075.068	9.201832	0.005679	4.066181	Not significant
TT	Between Groups	442.366	3	147.4553	50.07865	1.57E-05	4.066181	significant
YM	Between Groups	0.244815	3	0.081605	40.06061	3.64E-05	4.066181	significant

SS= sum of square, df=degree of freedom, MS=mean square, F=F-value
